# Nodular Lymphocyte Predominant Hodgkin Lymphoma and T Cell/Histiocyte Rich Large B Cell Lymphoma - Endpoints of a Spectrum of One Disease?

**DOI:** 10.1371/journal.pone.0078812

**Published:** 2013-11-11

**Authors:** Sylvia Hartmann, Claudia Döring, Christina Jakobus, Benjamin Rengstl, Sebastian Newrzela, Thomas Tousseyn, Xavier Sagaert, Maurilio Ponzoni, Fabio Facchetti, Chris de Wolf-Peeters, Christian Steidl, Randy Gascoyne, Ralf Küppers, Martin-Leo Hansmann

**Affiliations:** 1 Dr. Senckenberg Institute of Pathology, Goethe University, Frankfurt am Main, Germany; 2 Department of Pathology, University Hospitals K.U.Leuven, Leuven, Belgium; 3 Unit of lymphoid malignancies, Department of Pathology, Scientific Institute San Raffaele, Milan, Italy; 4 Department of Pathology, University of Brescia, Brescia, Italy; 5 Department of Pathology and Laboratory Medicine and the Centre for Lymphoid Cancer, British Columbia Cancer Agency, University of British Columbia, Vancouver, Canada; 6 Institute of Cell Biology (Cancer Research), Faculty of Medicine, University of Duisburg-Essen, Essen, Germany; University of Navarra, Center for Applied Medical Research, Spain

## Abstract

In contrast to the commonly indolent clinical behavior of nodular lymphocyte predominant Hodgkin lymphoma (NLPHL), T cell/histiocyte rich large B cell lymphoma (THRLBCL) is frequently diagnosed in advanced clinical stages and has a poor prognosis. Besides the different clinical presentations of these lymphoma entities, there are variants of NLPHL with considerable histopathologic overlap compared to THRLBCL. Especially THRLBCL-like NLPHL, a diffuse form of NLPHL, often presents a histopathologic pattern similar to THRLBCL, suggesting a close relationship between both lymphoma entities. To corroborate this hypothesis, we performed gene expression profiling of microdissected tumor cells of NLPHL, THRLBCL-like NLPHL and THRLBCL. In unsupervised analyses, the lymphomas did not cluster according to their entity. Moreover, even in supervised analyses, very few consistently differentially expressed transcripts were found, and for these genes the extent of differential expression was only moderate. Hence, there are no clear and consistent differences in the gene expression of the tumor cells of NLPHL, THRLBCL-like NLPHL and THRLBCL. Based on the gene expression studies, we identified BAT3/BAG6, HIGD1A, and FAT10/UBD as immunohistochemical markers expressed in the tumor cells of all three lymphomas. Characterization of the tumor microenvironment for infiltrating T cells and histiocytes revealed significant differences in the cellular composition between typical NLPHL and THRLBCL cases. However, THRLBCL-like NLPHL presented a histopathologic pattern more related to THRLBCL than NLPHL. In conclusion, NLPHL and THRLBCL may represent a spectrum of the same disease. The different clinical behavior of these lymphomas may be strongly influenced by differences in the lymphoma microenvironment, possibly related to the immune status of the patient at the timepoint of diagnosis.

## Introduction

**Figure 1:**
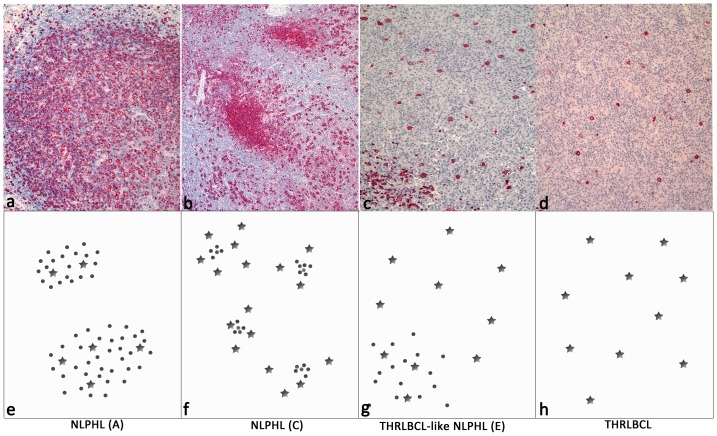
Immunoarchitectural patterns of NLPHL, THRLBCL-like NLPHL and THRLBCL, modified after Fan et al.[5]. a.–d. CD20-immunostainings (100x) of NLPHL patterns A and C, THRLBCL-like NLPHL and THRLBCL. e.–h. Schematic forms of immunoarchitectural patterns. Stars: tumor cells, dots: reactive B cells. a./e. Typical NLPHL Fan pattern A; b./f. NLPHL Fan pattern C; c./g. THRLBCL-like NLPHL (Fan pattern E); d./h. THRLBCL.

Nodular lymphocyte predominant Hodgkin lymphoma (NLPHL) is a germinal center (GC) B cell derived neoplasm preferentially affecting young to middle aged male patients [1], [2]. Diagnosis of NLPHL often reveals a limited stage disease with an indolent clinical behavior [3]. In most cases the histopathologic picture of NLPHL is dominated by a nodular infiltrate composed of small reactive B cells and only few tumor cells, the lymphocyte predominant (LP) cells [4]. However, cases of NLPHL have been described showing a diffuse infiltrate of LP cells in a T cell and histiocyte-rich background [5], [6]. Six NLPHL variant patterns have been defined by Fan et al., of which the patterns C and E most closely resemble T cell/histiocyte rich large B cell lymphoma (THRLBCL) (Fig. 1) [5]. Patients with NLPHL pattern E (in the following called THRLBCL-like NLPHL) develop relapses more frequently than patients with a typical nodular infiltrate [5]. 60% of these rare THRLBCL-like NLPHL cases present with advanced clinical stages (III/IV) [6].

THRLBCL is an aggressive B cell lymphoma and has been recognized as a new entity in the WHO classification of tumors of hematopoietic and lymphoid tissue [4]. It usually presents in advanced clinical stages and patients affected are usually middle aged males [7]. Some studies reported a poor clinical outcome [7], [8], whereas others found overall survival comparable to conventional diffuse large B cell lymphoma (DLBCL) [9]. Nonetheless, prognosis of THRLBCL is worse than for NLPHL [10]. The histopathologic picture of THRLBCL is dominated by a diffuse T cell and histiocyte-rich infiltrate comprising only few tumor cells [11]. Interestingly, there is a considerable diagnostic overlap between THRLBCL and THRLBCL-like NLPHL. The WHO classification [4] proposes to label cases with at least one typical NLPHL nodule as THRLBCL-like NLPHL and to distinguish these cases from primary THRLBCL.

The present study was aimed to clarify whether NLPHL and THRLBCL as well as THRLBCL-like cases can be clearly differentiated by global gene expression profiling (GEP) of the tumor cells or the composition of the reactive background.

## Materials and Methods

### Patient Selection

Cases of all patients analyzed by GEP were selected and reviewed by a hematopathologist panel (R.G., M.L.H., S.H., T.T.). THRLBCL-like NLPHL cases mostly resembled the morphology of THRLBCL, but at least one typical nodule of NLPHL was found. In the THRLBCL cases no coexisting NLPHL was found. Of the typical NLPHL cases, 8 of 10 were histologically classified as pattern A or B, and two cases were classified as pattern F, according to Fan et al. [5]. Cases were collected at the Dr. Senckenberg Institute of Pathology Frankfurt am Main, Germany, the Department of Pathology University Hospitals K.U.Leuven, Belgium, the Unit of lymphoid malignancies Scientific Institute San Raffaele, Milan, Italy, the Department of Pathology University of Brescia, Italy and the Department of Pathology and Laboratory Medicine and the Centre for Lymphoid Cancer British Columbia Cancer Agency, Vancouver, Canada. The local ethics committees approved the study and written informed consent from the donors was obtained in accordance with the Declaration of Helsinki.

Clinical data of cases investigated by GEP are provided in Table S1. The tumor cells in all cases were positive for CD20 and negative for CD30, CD15 and Epstein-Barr virus (EBER). An independent series of 10 lymph nodes each from patients with NLPHL (Fan patterns A and C), THRLBCL-like NLPHL (NLPHL pattern E), and THRLBCL were investigated by immunohistochemistry for confirmation purposes.

### Purification of Cells

Twohundredtwenty tumor cells from fresh frozen lymph nodes of 10 cases of typical NLPHL (pattern A/B/F,. Methods S1), 9 cases of THRLBCL-like NLPHL (pattern E) and 11 cases of THRLBCL were laser microdissected as previously described [12]. For comparison to tumor cells, CD77^+^ normal GC B cells were purified from fresh tonsils of healthy donors using magnetic activated cell sorting (MACS; Miltenyi Biotech, Bergisch Gladbach, Germany), as reported previously [13].

### Gene Expression Analysis

Microdissected and MACS purified cells were lysed in NUGEN Direct Lysis Buffer and total RNA was amplified with the WT-Ovation-One-direct-Kit according to the manufactureŕs protocol (NUGEN, Bemmel, The Netherlands). The samples were labeled with the Encore Biotin Module (NUGEN) and hybridized onto Human Gene 1.0 ST Arrays (Affymetrix, Santa Clara, USA). Detailed information on sample purification and statistical analysis can be found in Methods S1 section. Gene expression data are available through the GEO database (GSE47044).

### Immunohistochemistry

Results of gene expression profiling were confirmed by immunohistochemistry as previously described [14]. In brief, paraffin sections of an independent series of 10 lymph nodes each from patients with NLPHL (patterns A and C), THRLBCL-like NLPHL (NLPHL pattern E), and THRLBCL were assessed for BAG6/BAT3, HIGD1A, UBD/FAT10 and CXCL13 expression. Six reactive lymph nodes were likewise stained for BAG6/BAT3, HIGD1A and UBD/FAT10. All cases were additionally stained for CD163, CD4, CD8 and MUM1, as well as the follicular dendritic cell (FDC) marker CD21 and follicular T helper (T_FH_) markers PD1, CXCL13 and ICOS as described previously [15], [16]. Antibodies and staining procedures are summarized in Table S2.

### Quantification of Tumor Microenvironment

Immunohistochemical CD4-, CD8- and CD163-stainings of the cases investigated by gene expression profiling and of additional independent cases were quantified using a light microscope equipped with a camera system (Microdissection Axiovert 200M microscope, PALM, Bernried, Germany). For each slide, three representative counting frames (122,824 µm^2^) were chosen, and the number of positively stained cells was point-counted using PALM software as described previously [15]. In tumors with a nodular infiltrate counting frames were placed in the nodules, in predominantly diffuse infiltrates, counting frames were set in the diffuse areas. Cell counting and assessment of histological parameters were carried out in each case without knowledge of the diagnosis and clinical parameters. The data were tested for the presence of a Gaussian distribution (Shapiro-Wilk-test). Significance was calculated using either unpaired two-tailed Students t-test or Mann-Whitney-test.

The percentage of PD1^+^, CXCL13^+^ and ICOS^+^ T_FH_ cells was estimated to be <5%, or 10–90% in steps of 10%. The pattern of FDCs in CD21 staining was classified as large nodules, small nodules or absent.

## Results

### Gene Expression Profiles of the Tumor Cells of NLPHL and THRLBCL are Closely Related

**Figure 2:**
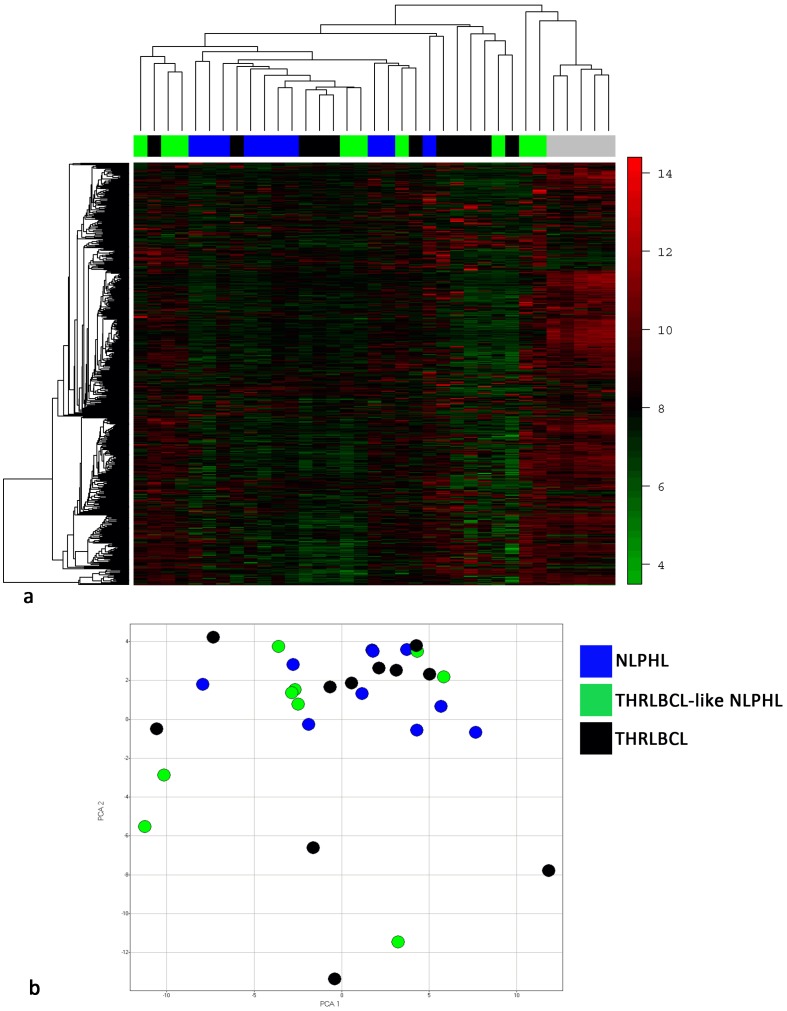
Unsupervised hierarchical clustering and principal component analysis of gene expression profiles of microdissected tumor cells of NLPHL, THRLBCL-like NLPHL and THRLBCL, as well as sorted tonsillar GC B cells. **a.** Unsupervised hierarchical clustering of the gene expression profiles of the tumor cells of NLPHL, THRLBCL-like NLPHL and THRLBCL as well as CD77^+^ GC B cells. All probesets with a standard deviation ≥0.9 (479 probesets) were considered. GC B cell samples are indicted by a grey bar. **b.** Principal component analysis of the gene expression profiles of the tumor cells of NLPHL, THRLBCL-like NLPHL and THRLBCL. All probesets with a standard deviation ≥1.2 (79 probesets, 40.18% diversity) were considered.

To elucidate the relatedness of the tumor cells of NLPHL, THRLBCL-like NLPHL and THRLBCL, we generated global gene expression profiles of microdissected tumor cells from 10 cases of NLPHL, 9 cases of THRLBCL-like NLPHL, and 11 cases of THRLBCL, as well as 5 samples of MACS-isolated tonsillar GC B cells. We performed an unsupervised hierarchical clustering of the gene expression profiles of all cases together with the GC B cells (Fig. 2a). A core group of 7 of 10 typical NLPHL was identified that clustered closely together. However, this group did not cluster completely separately from the rest of the cases. Instead, there was a continuous branching, and THRLBCL-like NLPHL cases were intermingled with THRLBCL cases. The GC B cells clustered in a separate group. Performing unsupervised hierarchical clustering without GC B cells, a similar pattern with continuous branching of the tumor cases was observed (Fig. S1). In a principal component analysis (Fig. 2b), similar results were obtained: the tumor cells of the single cases of each entity were heterogeneously distributed and again the different entities did not clearly separate.

### Comparison of Tumor Cells of NLPHL and THRLBCL Highlights Only Few Differentially Expressed Genes

In a supervised comparison of the global gene expression patterns of the tumor cells from the NLPHL, THRLBCL-like NLPHL and THRLBCL included in our analysis no differentially expressed genes were identified if a false discovery rate (FDR) below 0.1 was applied. After relaxing the FDR to 0.3, allowing a rate of 30% false positive discoveries, some differentially expressed genes were obtained. Nevertheless, no differentially expressed genes were detected between typical NLPHL and the group of THRLBCL-like NLPHL. Solely one differentially expressed gene was found between THRLBCL-like NLPHL and THRLBCL (HIGD1A, a hypoxia-inducible target gene [17], 2.4-fold upregulated in THRLBCL-like NLPHL, FDR = 0.104, p<0.0001; Table 1). In the comparison of LP cells of typical NLPHL with tumor cells of THRLBCL, the highest number of differentially expressed genes was observed; 16 genes were upregulated more than 1.7-fold in the LP cells of typical NLPHL, including HIGD1A (upregulated 2.0-fold, Table 1, Table S3). Another upregulated gene was SEPT14, a GTP-binding cytoskeletal protein implicated in multiple cellular functions, including apoptosis, cell polarity, cell cycle regulation, and oncogenesis [18]. Further upregulated genes were a regulator of G protein signaling (RGS13), amylase alpha 2A (AMY2A) and ribosomal proteins (RPS27 and MRPL51). Among the 8 genes upregulated in the tumor cells of THRLBCL compared to LP cells of typical NLPHL (Table 1, Table S4), BAT3/BAG6 was most strongly upregulated (3.5-fold). BAT3/BAG6 is a protein with multiple functions, including acetylation of p53 [19], degradation of mislocalized proteins [20] and regulation of apoptosis [21].

**Table 1:** Top 5 upregulated genes in pairwise comparisons between tumor cells of NLPHL, THRLBCL-like NLPHL and THRLBCL.

Comparison	Top-5 Genes	Fold change	FDR	
NLPHL vs THRLBCL	SEPT14	2.7	0.27	Septin 14
	RGS13	2.3	0.21	Regulator of G-Protein Signalling 13
	AMY2A	2.2	0.21	Amylase alpha 2A
	SNORD75	2.2	0.29	Small nucleolar RNA 75
	RPS27	2.2	0.21	Ribosomal protein S27
NLPHL vs THRLBCL-like NLPHL	–	–	–	–
THRLBCL vs NLPHL	BAT3	3.5	0.27	HLA-B associated transript 3
	MT2A	3.1	0.24	Metallothionein 2A
	MT1H	3.1	0.21	Metallothionein 1H
	CXCL9	2.3	0.22	Chemokine ligand 9
	S100A8	2.1	0.22	S100 Calcium-binding protein A8
THRLBCL-like NLPHL vs NLPHL	–	–	–	–
THRLBCL vs THRLBCL-like NLPHL	–	–	–	–
THRLBCL-like NLPHL vs THRLBCL	HIGD1A	2.4	0.10	HIG hypoxia inducible domain family member 1A

p-values <0.05. FDR (false discovery rate)<0.3. vs = versus.

In the supervised comparisons with GC B cells (p<0.05, FDR <0.1, Fold change >1.7), 44 genes were upregulated in the LP cells of typical NLPHL (Table S5), 40 genes in the LP cells of THRLBCL-like NLPHL (Table S6) and 28 genes in the tumor cells of THRLBCL (Table S7). Of note, ubiquitin-like modifier UBD/FAT10, which is modifying p53 [22], mediating NF-kappaB activation [23], and can be induced by pro-inflammatory stimuli [24], was particularly highly upregulated. CXCL13, expressed in follicular T helper cell derived lymphomas [25], was 1.7- and 1.9-fold upregulated in LP cells of typical NLPHL and THRLBCL-like NLPHL, respectively, as well as 1.5-fold in tumor cells of THRLBCL.

### Identification of Novel Immunohistochemical Markers

**Figure 3:**
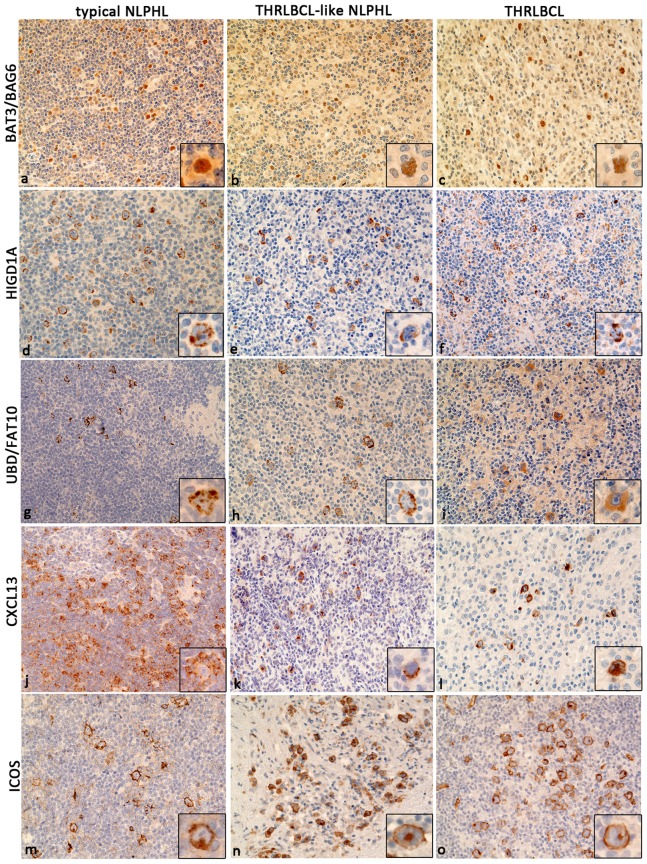
Validation on protein level of genes expressed in both THRLBCL-like NLPHL and THRLBCL identified by gene expression profiling. **a., b. and c.:** Strong expression of BAT3/BAG6 in the tumor cells of typical NLPHL (pattern A), THRLBCL-like NLPHL and THRLBCL, 200x. Inset: Positive tumor cells in 400x. **d., e. and f.:** Expression of HIGD1A in the tumor cells of typical NLPHL (pattern A), THRLBCL-like NLPHL and THRLBCL, 200x. Inset: Positive tumor cells in 400x. **g., h. and i.:** Expression of UBD/FAT10 in the tumor cells of typical NLPHL (pattern A), THRLBCL-like NLPHL and THRLBCL, 200x. Inset: Positive tumor cells in 400x. **j., k. and l.:** Expression of CXCL13 in rosetting T cells of typical NLPHL (pattern A) and in the tumor cells of THRLBCL-like NLPHL and THRLBCL, 200x. Insets in 400x. **m., n. and o.:** Expression of ICOS in the tumor cells of typical NLPHL (pattern A), THRLBCL-like NLPHL and THRLBCL, 200x. Inset: Positive tumor cells in 400x.

Immunohistochemical stainings for the proteins HIGD1A, BAT3/BAG6, UBD/FAT10 and CXCL13, identified by gene expression profiling, were performed on independent cases of typical NLPHL (patterns A and C), THRLBCL-like NLPHL (pattern E) and THRLBCL. HIGD1A expression was detected more frequently and stronger in tumor cells of different variants of NLPHL (6/10, 8/10 and 9/10 cases, respectively in the patterns A, C and E) than in THRLBCL (4/10 cases, Table 2, Fig. 3). In contrast, a strong expression of BAT3/BAG6 was found in tumor cells of THRLBCL (10/10 cases, Fig. 3), but a somewhat weaker expression was also observed in different variant patterns of NLPHL (10/10 and 8/10 cases for patterns A and C, respectively) and THRLBCL-like NLPHL (7/10 cases). UBD/FAT10 was rarely expressed in typical NLPHL (1/10 cases each for patterns A and C), but was more frequently expressed in THRLBCL-like NLPHL (5/10 cases) and THRLBCL (4/10). HIGD1A and BAT3/BAG6 were also weakly expressed in a subset of GC B cells in reactive lymph nodes, whereas UBD/FAT10 was negative. CXCL13 was expressed in the tumor cells in a small subset of cases of NLPHL variants with predominant extranodular localization of the tumor cells (4/10 and 2/10 cases, respectively, for NLPHL pattern C and THRLBCL-like NLPHL) and in THRLBCL (2/10 cases), consistent with the weak upregulation observed in tumor cells compared to the GC B cells in the gene expression profiling.

**Table 2:** Immunohistochemical validation of genes expressed in the tumor cells of NLPHL and THRLBCL in an independent set of cases.

	HIGD1A	BAT3	FAT10	CXCL13	ICOS
Typical NLPHL(Pattern A)	6/10	10/10	1/10	0/10	1/10
Typical NLPHL(Pattern C)	8/10	8/10	1/10	4/10	0/10
THRLBCL-likeNLPHL (Pattern E)	9/10	7/10	5/10	2/10	5/10
THRLBCL	4/10	10/10	4/10	2/10	1/10

### Quantification of the Microenvironment Reveals Differences between Typical NLPHL and THRLBCL, and Similarities between THRLBCL-like NLPHL and THRLBCL

**Figure 4:**
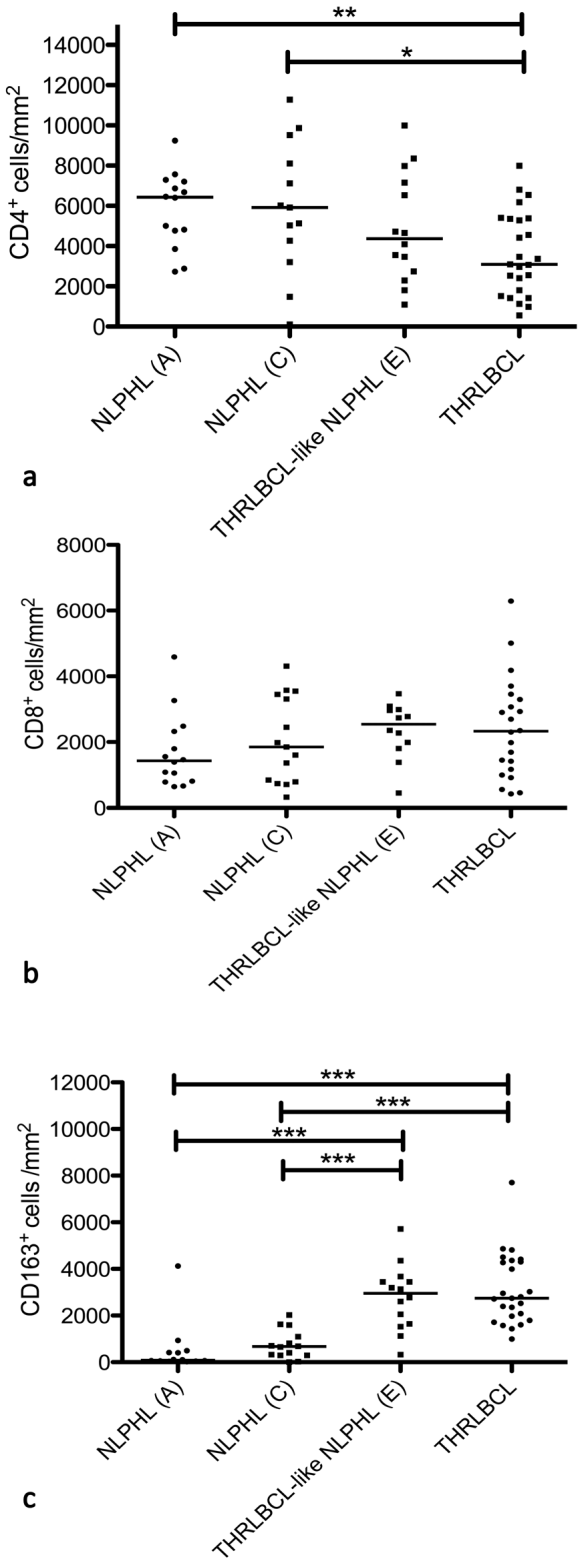
Quantification of the microenvironment in typical NLPHL (patterns A and C), THRLBCL-like NLPHL (pattern E) as well as THRLBCL. **a.** Numbers of CD4^+^ T cells/mm^2^ in typical NLPHL (pattern A: n = 14 and pattern C: n = 13), THRLBCL-like NLPHL (n = 14) and THRLBCL (n = 25). (*p<0.05, **p<0.01, unpaired t-test). **b.** Numbers of CD8^+^ T cells/mm^2^ in typical NLPHL (pattern A: n = 14, pattern C: n = 15) and THRLBCL-like NLPHL (n = 12) as well as THRLBCL (n = 22). **c.** Numbers of CD163^+^ macrophages/mm^2^ in NLPHL (pattern A and C as well as THRLBCL-like NLPHL: n = 14, each) and THRLBCL (n = 25), (***p<0.001, Mann-Whitney-test).

Since gene expression profiling of tumor cells did not reveal clear differences between NLPHL and THRLBCL, we aimed to further characterize the microenvironment of the different entities in order to evaluate the importance of the microenvironment. It is well known that reactive T cells in THRLBCL mainly correspond to CD8^+^ T cells with only few CD4^+^ T cells [26], [27]. Therefore, we determined the number of CD4^+^ and CD8^+^ T cells as well as CD163^+^ histiocytes in histological sections of NLPHL and THRLBCL. CD4^+^ T-cell counts in THRLBCL (median 3091/mm^2^) were significantly lower than in typical NLPHL (pattern A: median 6433/mm^2^, p = 0.002, t-test; pattern C: 5966/mm^2^, p = 0.011, t-test; Fig. 4a). The difference in CD4^+^ T-cell amount of THRLBCL-like NLPHL (median 4374/mm^2^) compared to typical NLPHL was not significant. Furthermore, CD8^+^ T-cell counts did not significantly differ between typical NLPHL, THRLBCL-like NLPHL and THRLBCL (Fig. 4b). In addition, the amount of CD163^+^ histiocytes was determined. The average number of CD163^+^ histiocytes/mm^2^ was significantly higher in both THRLBCL-like NLPHL and THRLBCL (median 2957 and 2743/mm^2^, respectively) compared to typical NLPHL (pattern A and C median 85 and 689/mm^2^, p<0.001, Mann-Whitney-test, Fig. 4c).

In ten cases each, the percentage of reactive T cells expressing the T_FH_ cell markers PD1, CXCL13 and ICOS was assessed. Generally, typical NLPHL (patterns A and C) showed a higher content of T_FH_ cells compared to THRLBCL-like NLPHL and THRLBCL (Fig. S2). PD1^+^ T cells were slightly more frequent (medians 30% in NLPHL pattern A, 35% in pattern C, 15% in THRLBCL-like NLPHL and 5% in THRLBCL) compared to CXCL13^+^ T cells (medians 20% in NLPHL pattern A, 15% in pattern C, 7.5% in THRLBCL-like NLPHL and 5% in THRLBCL) and ICOS^+^ T cells (medians 20% in NLPHL pattern A, 25% in pattern C and 5% each in THRLBCL-like NLPHL and THRLBCL).

In previous studies, CD57^+^ and PD1^+^ T cells rosetting around tumor cells were frequently observed in typical NLPHL, but less often in THRLBCL-like NLPHL [28], [29]. We therefore studied the cases investigated also for the presence of rosetting T cells expressing PD1, ICOS, CXCL13 and MUM1 (Table 3). PD1^+^ rosetting T cells were frequently observed in typical NLPHL (pattern A: 8/10 cases and pattern C: 6/10 cases), whereas no PD1^+^ rosetting T cells were found in THRLBCL-like NLPHL and THRLBCL. Likewise, MUM1^+^ T cell rosettes were more frequently observed in typical NLPHL (pattern A and C: 6/10 cases each) than in THRLBCL-like NLPHL and THRLBCL, where they were present in 2/10 cases each. CXCL13^+^ and ICOS^+^ rosetting T cells were only rarely observed in typical NLPHL (Table 3). Surprisingly, in the ICOS stainings, few NLPHL and THRLCBL cases could be identified, with tumor cells expressing ICOS (Table 2, Fig. 3). The majority of these cases (5/7) belonged to the THRLBCL-like NLPHL group. However, one ICOS^+^ case each was also found in the NLPHL pattern A and THRLBCL group. Interestingly, in all these cases the content of reactive ICOS^+^ T cells was very low (in all cases ≤10%). We also evaluated the gene expression data for ICOS and ICOS-ligand (ICOSLG) expression in the different lymphoma subsets, assuming cases with log_2_ expression values ≥7.0 to show an expression of these transcripts: ICOS was expressed in this independent set of cases only in rare cases (3 cases typical NLPHL, 1 case THRLBCL-like NLPHL, 2 cases THRLBCL), whereas ICOSLG was expressed in all cases as well as in the GC B cells.

**Table 3:** Immunophenotype of rosetting T cells in different variants of NLPHL and THRLBCL*.

	MUM1^+^ rosetting T cells	PD1+ rosetting T cells	CXCL13+ rosetting T cells	ICOS+ rosetting T cells
Typical NLPHL (Pattern A)	6/10	8/10	3/10	1/10
Typical NLPHL (Pattern C)	6/10	6/10	0/10	1/10
THRLBCL-like NLPHL (Pattern E)	2/10	0/10	0/10	0/10
THRLBCL	2/10	0/10	0/10	0/10

* Cases were scored positive if positive rosetting T cells were observed around at least 5% of the tumor cells.

The FDC content in the cases correlated very well with the patterns studied: 9/10 NLPHL pattern A presented large FDC nodules and one case presented small nodules. In 6/10 NLPHL pattern C small FDC nodules dominated, whereas four cases showed large nodules. In both the diffuse areas of THRLBCL-like NLPHL as well as in the THRLBCL cases remnants of FDC were not identified.

## Discussion

In the present study we performed gene expression profiling of microdissected LP cells of different variants of NLPHL (typical and THRLBCL-like NLPHL) in comparison to tumor cells of THRLBCL. Applying this method, we found an important molecular overlap between the tumor cells of the different variants of NLPHL and THRLBCL. We cannot exclude that this result is influenced by technical limitations, like a strong amplification of the RNA before array hybridization, causing loss of some differentially expressed genes. However, applying the same technique, we obtained numerous and up to 21-fold differentially expressed genes in another study of microdissected cells [12]. Both the unsupervised hierarchical clustering as well as the principal component analysis revealed that there is often more heterogeneity in global gene expression between two cases of one of the three lymphoma entities analyzed than between cases of different entities. Even in the pairwise supervised comparisons of NLPHL, THRLBCL-like NLPHL and THRLBCL, very few consistently differentially expressed genes were found. Thus, no indication was found that the tumor cells of the three types of lymphomas show a disease-specific gene expression pattern, which would have supported a distinct pathogenesis and pathophysiology of the tumor cells.

The lack of clear distinct gene expression patterns between NLPHL and THRLBCL as seen in our analysis is in line with results of other studies, in which it was not possible to discriminate both entities on the basis of differential marker expression in the tumor cells [11], [13], [30]. Indeed, a previously published gene expression study comparing the gene expression profiles of tumor cells of different B cell lymphomas reported that tumor cells of THRLBCL were most closely related to tumor cells of typical nodular NLPHL [13]. However, THRLBCL-like NLPHL was not included in the analysis, nor in other studies, which aimed to identify differentially expressed markers between the two entities [30], [31], [32], [33]. So far, many commonly expressed genes in tumor cells of both entities were identified, such as BCL6, CD75, EMA, J-chain and PU.1, as well as a typical kappa light chain restriction [11], [34], [35], [36], [37]. The only investigations which clearly point to a distinct pathogenesis of both diseases are two comparative genomic hybridization studies, in which a higher number and different genomic aberrations were identified in NLPHL than in THRLBCL [38], [39]. However, these distinct genetic patterns apparently do not translate into many consistently differentially expressed genes, as implied from the present work.

The genes we identified to be expressed in typical NLPHL, THRLBCL-like NLPHL and THRLBCL, were ubiquitin-like modifiers like UBD/FAT10 and BAT3/BAG6, both interacting with the tumor suppressor p53 [19], [22]. In a previous study, p53 expression was observed in 72% of the tumor cells of THRLBCL [11]. This is surprising, since BCL6 has been shown to downregulate p53 [40], and BCL6 is strongly expressed in the tumor cells of NLPHL, THRLBCL-like NLPHL and THRLBCL [11], [41]. It has been shown that BAT3/BAG6 can be released from tumor cells in response to stress signals [42]. Both BAT3/BAG6 as well as UBD/FAT10 expression was shown to be increased during inflammatory response [21], [43]. This may be caused by the release of proinflammatory factors from macrophages of THRLBCL [12]. Moreover, HIGD1A, an important player in the maintenance of normal mitochondrial function in hypoxia [17], [44], [45], was expressed in a subset of cases. Thus, the genes expressed in tumor cells of both entities hint at a severe stress, possibly related to a proinflammatory milieu created by macrophages and epithelioid cells. Expression of CXCL13 was only rarely observed in tumor cells of THRLBCL-like NLPHL as well as THRLBCL, but never in typical NLPHL (pattern A). Instead, 3 out of 10 NLPHL pattern A cases presented CXCL13-positive rosetting T cells (Fig. 3). It might be speculated that CXCL13-expression supports independency of the tumor cells from FDC networks and CXCL13-positive rosetting T cells [28], [46], which are both absent in THRLBCL-like NLPHL and THRLBCL. A similar observation was made for ICOS expression in the tumor cells of one case each of typical NLPHL and THRLBCL as well as in five cases of THRLBCL-like NLPHL. If aberrant ICOS expression in the tumor cells occurred, only few ICOS^+^ T_FH_ cells were found in the reactive microenvironment. Therefore, an autocrine stimulation of the tumor cells via ICOSLG could be a possible explanation, particularly since in the gene expression analysis ICOSLG was expressed in the tumor cells of all NLPHL and THRLBCL cases. Since the majority of typical NLPHL usually include a relatively high proportion of ICOS^+^ T_FH_ cells, LP cells would not benefit from aberrant ICOS expression and therefore LP cells usually appear to be ICOS^−^ [47].

In a previous whole tissue gene expression study, large differences in the gene expression of the microenvironment of typical NLPHL and THRLBCL were found [48]. In the present study, we confirmed differences in the composition of the microenvironment of typical NLPHL (pattern A and C) and THRLBCL in terms of T_FH_ cell and macrophage content. Reduced numbers of T_FH_ cells in diffuse variants of NLPHL have previously been described [28], [29]. However, we could not demonstrate clear differences between THRLBCL-like NLPHL and THRLBCL. Furthermore, MUM1^+^ rosetting T cells were present in rare cases of both THRLBCL-like NLPHL and THRLBCL. As patients were described to present with NLPHL and THRLBCL and vice versa in sequential biopsies [10], it seems very unlikely that the tumor cells in THRLBCL should be fundamentally different from NLPHL. Indolent Non-Hodgkin lymphomas frequently progress into aggressive B cell lymphomas, but very rarely primary aggressive B cell lymphomas relapse as an indolent B cell lymphoma, without a previous history of indolent Non-Hodgkin lymphoma. Therefore, we speculate that THRLBCL-like NLPHL and THRLBCL represent the same disease and may be an aggressive variant of NLPHL with unique changes in the microenvironment.

In recent studies, the composition of the microenvironment and particularly a high content of tissue macrophages was identified to be an adverse prognostic factor in classical Hodgkin lymphoma (cHL) [49], [50], [51], [52]. Furthermore, not only the composition of the microenvironment in the tissue, but additionally a low ratio of absolute lymphocyte/monocyte count in the peripheral blood was identified as an independent prognostic factor for overall and progression free survival in patients with cHL and NLPHL [53], [54], [55]. We recently demonstrated a link between low CD4^+^ blood counts and a low amount of CD4^+^ T cells in combination with a high content of macrophages in the tissues affected by cHL in HIV patients [15]. Although the amounts of CD4^+^ T cells in THRLBCL and THRLBCL-like NLPHL were not comparable to the setting of HIV-associated cHL, they were significantly decreased in THRLBCL compared to typical NLPHL. The macrophage counts in THRLBCL and THRLBCL-like NLPHL were in the range of HIV-associated cHL [15]. One possible hypothesis to explain this observation could be that monocytes/macrophages are attracted by the tumor cells to the affected lymph nodes via CCL5 [56], [57] in the absence or relative lack of CD4^+^ T cells in the blood. In the present study, CCL5 was upregulated in all tumor samples compared to GC B cells, but the upregulation was significant only in THRLBCL tumor cells (1.5-fold upregulation, FDR = 0.037, p = 0.014, t-test). We therefore propose that the manifestation of typical NLPHL or THRLBCL-like NLPHL/THRLBCL may depend on the ratio of CD4^+^ T cells/monocytes in the blood of the patient at diagnosis. Similar to HIV-patients with cHL [58], THRLBCL-like NLPHL and THRLBCL patients generally present with advanced disease stages [7]. In the present study, we cannot finally clarify the relationship between NLPHL and THRLBCL. However, since the clinical presentation of the patients with both diseases is distinct, requiring different treatment approaches, it appears practical to furtherhin separate patients in the two diagnostic categories of NLPHL and THRLBCL. Our observation of a highly similar immunophenotype of the tumor cells in both entities together with a remarkably different composition of the microenvironment suggest a fundamental role of the patients immune status at diagnosis. Further studies, particularly investigating common and distinct genomic aberrations and the immune status at diagnosis, will be necessary to corroborate this hypothesis.

## Supporting Information

Figure S1 **Unsupervised hierarchical clustering.**
(TIF)

Figure S2 **Percentages of CXCL13-, PD1- and ICOS- positive cells.**
(TIF)

Table S1 Clinical characteristics of patients included in the gene expression analysis.
(DOC)

Table S2 Antibodies and dilutions applied for immunohistochemistry.
(DOC)

Table S3 All genes differentially upregulated in LP cells of typical NLPHL compared to tumor cells of THRLBCL.
(DOC)

Table S4 All genes differentially upregulated in tumor cells of THRLBCL compared to LP cells of typical NLPHL.
(DOC)

Table S5 All genes upregulated in LP cells of typical nodular NLPHL compared to GC B cells.
(DOC)

Table S6 All genes upregulated in LP cells of NLPHL pattern E compared to GC B cells.
(DOC)

Table S7 All genes upregulated in tumor cells of THRLBCL compared to GC B cells.
(DOC)

Methods S1 (DOC)
