# Supplementary figures and images for: Nodular Lymphocyte Predominant Hodgkin Lymphoma and T Cell/Histiocyte Rich Large B Cell Lymphoma - Endpoints of a Spectrum of One Disease?
Source: PLoS One. 2013 Nov 11;8(11):e78812. doi: 10.1371/journal.pone.0078812 (PMC3823948; doi:10.1371/journal.pone.0078812)

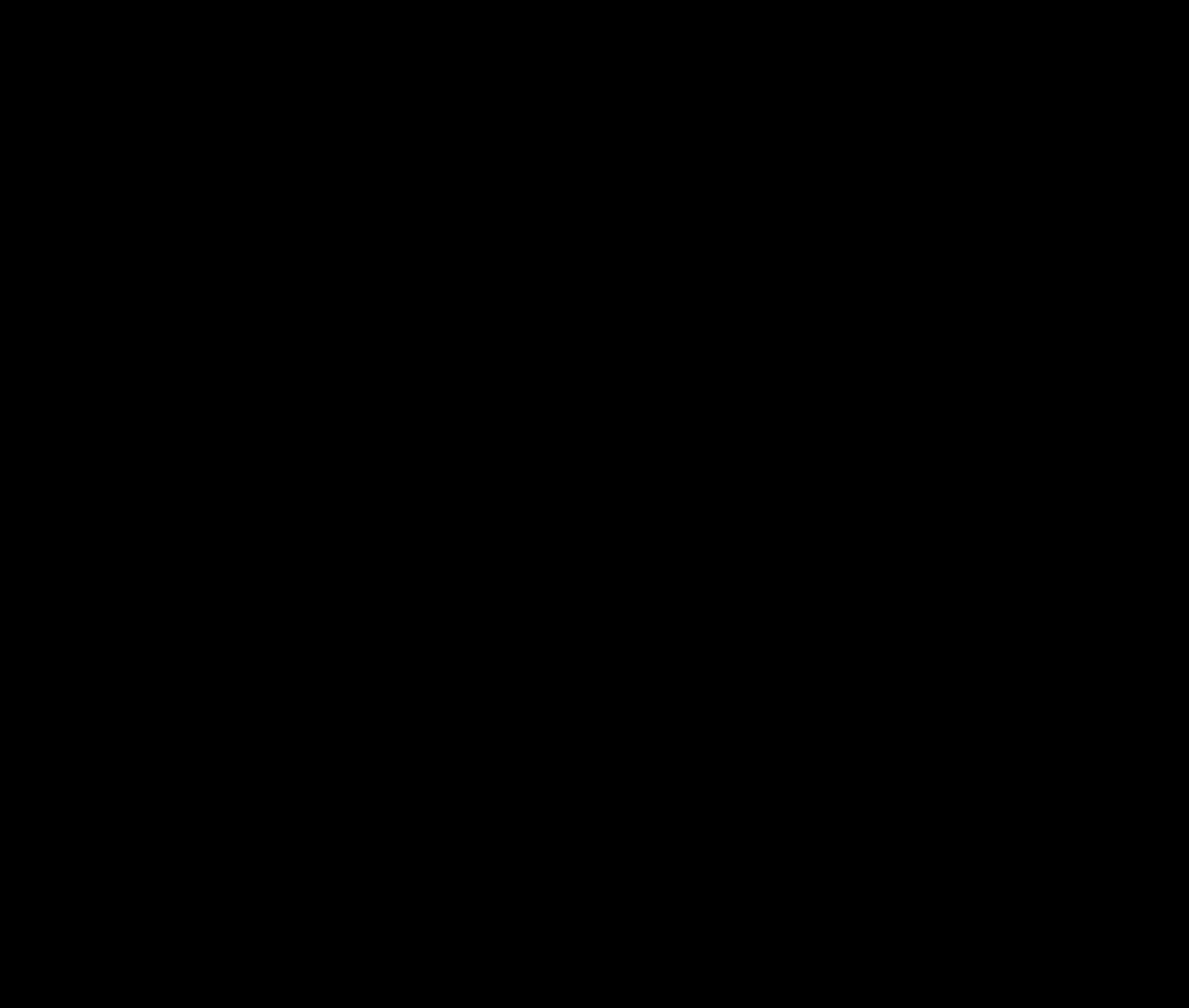

Supplement: Figure S1 — Unsupervised hierarchical clustering. (TIF) [file pone.0078812.s001.tif]

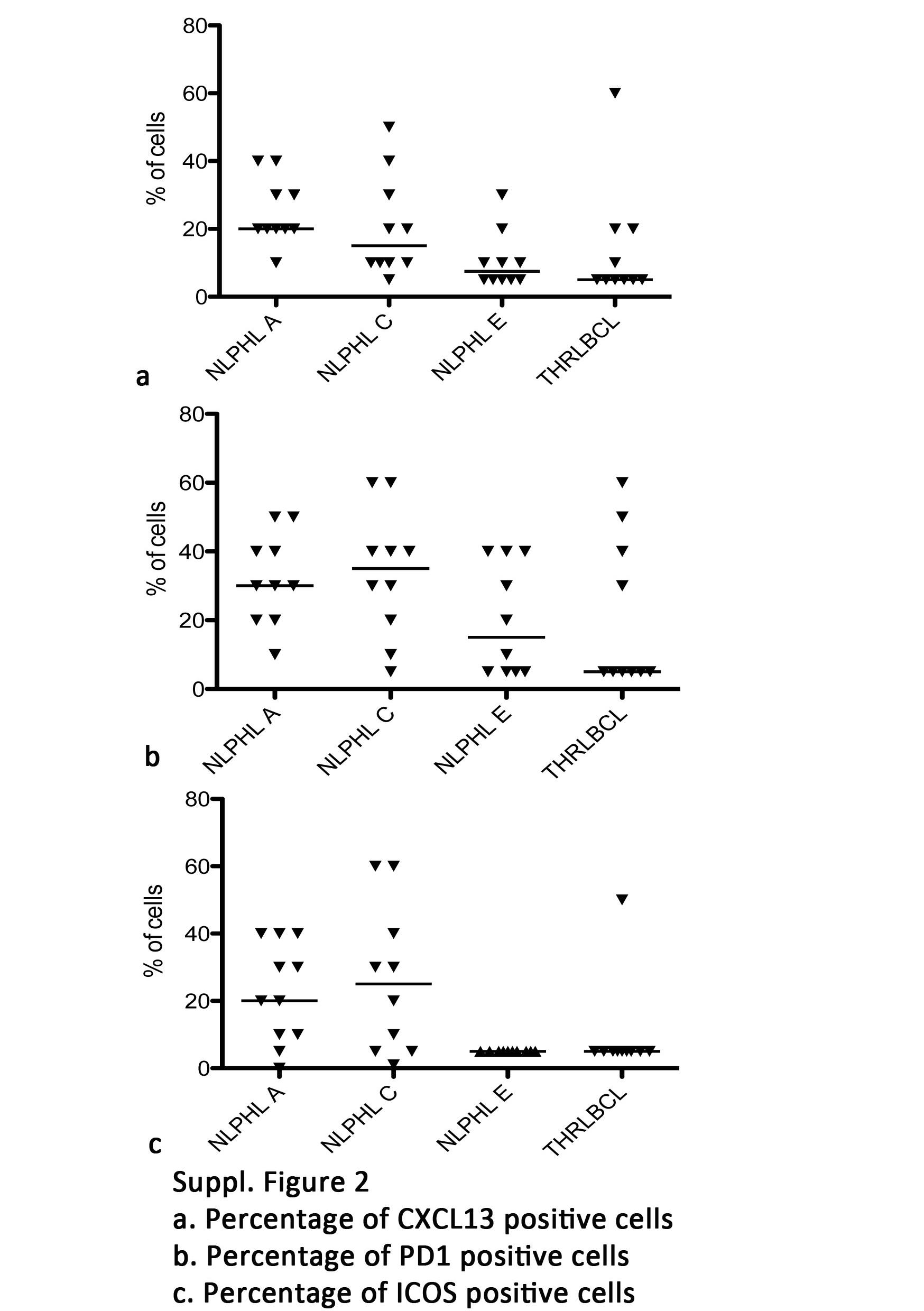

Supplement: Figure S2 — Percentages of CXCL13-, PD1- and ICOS- positive cells. (TIF) [file pone.0078812.s002.tif]
