# Supplementary material for: Nodular Lymphocyte Predominant Hodgkin Lymphoma and T Cell/Histiocyte Rich Large B Cell Lymphoma - Endpoints of a Spectrum of One Disease?
Source: PLoS One. 2013 Nov 11;8(11):e78812. doi: 10.1371/journal.pone.0078812 (PMC3823948; doi:10.1371/journal.pone.0078812)
Supplement: Table S1 — Clinical characteristics of patients included in the gene expression analysis. (DOC) [file pone.0078812.s003.doc]

|  | NLPHL | THRLBCL-like NLPHL | THRLBCL |
| --- | --- | --- | --- |
| No. of patients | 10 | 9 | 11 |
| Age, median in years | 46 | 46 | 42.5 |
| Age range (years) | 24-71 | 12-85 | 20-89 |
| Male gender (%) | 80 | 78 | 64 |
| Stage |  |  |  |
| - I | 5 | 1 |  |
| - II | 3 |  |  |
| - III | 1 | 4 | 2 |
| - IV |  | 3 | 7 |

Suppl. Table S1 Clinical characteristics of patients included in the gene expression analysis
