# Supplementary material for: Nodular Lymphocyte Predominant Hodgkin Lymphoma and T Cell/Histiocyte Rich Large B Cell Lymphoma - Endpoints of a Spectrum of One Disease?
Source: PLoS One. 2013 Nov 11;8(11):e78812. doi: 10.1371/journal.pone.0078812 (PMC3823948; doi:10.1371/journal.pone.0078812)
Supplement: Table S2 — Antibodies and dilutions applied for immunohistochemistry. (DOC) [file pone.0078812.s004.doc]

| Antibody | Dilution | Pretreatment | Provider |
| --- | --- | --- | --- |
| BAT3/BAG6 | 1:1000 | EDTA pH8 | Novus Biologicals, Cambridge, UK |
| HIGD1A | 1:50 | EDTA pH8 | Novus Biologicals, Cambridge, UK |
| FAT10/UBD | 1:1000 | EDTA pH8 | Novus Biologicals, Cambridge, UK |
| CXCL13 | 1:200 | pH9* | R&D Systems, Minneapolis, USA |
| ICOS | 1:250 | pH9* | R&D Systems, Minneapolis, USA |
| PD1 | 1:1000 | EDTA pH8 | Abcam, Cambridge, UK |
| CD21 | 1:100 | Proteinase K** | Dako, Glostrup, Denmark |
| MUM1 | 1:100 | EDTA pH8 | Dako, Glostrup, Denmark |

**Suppl. Table S2** Antibodies and dilutions applied for immunohistochemistry

* 10 min microwave, Target Retrieval Solution, high pH, DAKO

** 5 min incubation at room temperature, proteinase K S3020, DAKO
