# Supplementary material for: Nodular Lymphocyte Predominant Hodgkin Lymphoma and T Cell/Histiocyte Rich Large B Cell Lymphoma - Endpoints of a Spectrum of One Disease?
Source: PLoS One. 2013 Nov 11;8(11):e78812. doi: 10.1371/journal.pone.0078812 (PMC3823948; doi:10.1371/journal.pone.0078812)
Supplement: Table S4 — All genes differentially upregulated in tumor cells of THRLBCL compared to LP cells of typical NLPHL. (DOC) [file pone.0078812.s006.doc]

| Fold change | p-value | FDR | Gene Symbol | mRna - Description |
| --- | --- | --- | --- | --- |
| -1.2 | 0.126927356 | 0.451459666 | VWA1 | Homo sapiens von Willebrand factor A domain containing 1 (VWA1), transcript variant 1, mRNA. |
| -1.2 | 0.026236951 | 0.316836447 | MIB2 | Homo sapiens mindbomb homolog 2 (Drosophila) (MIB2), mRNA. |
| -1.2 | 0.05190056 | 0.351463158 | LOC100128003 | Homo sapiens hypothetical protein LOC100128003 (LOC100128003), non-coding RNA. |
| -1.3 | 0.034567482 | 0.326024849 | ESPN | Homo sapiens espin (ESPN), mRNA. |
| -1.1 | 0.389822781 | 0.686331196 | VAMP3 | Homo sapiens vesicle-associated membrane protein 3 (cellubrevin) (VAMP3), mRNA. |
| 1.1 | 0.522926407 | 0.765690978 | PARK7 | Homo sapiens Parkinson disease (autosomal recessive, early onset) 7 (PARK7), transcript variant 1, mRNA. |
| -1.2 | 0.306437332 | 0.626920532 | SLC25A33 | Homo sapiens solute carrier family 25, member 33 (SLC25A33), mRNA. |
| -1.1 | 0.599419703 | 0.812472643 | PGD | Homo sapiens phosphogluconate dehydrogenase (PGD), mRNA. |
| 3.5 | 0.010 | 0.265 | BAT3 | Homo sapiens HLA-B associated transcript 3 (BAT3), transcript variant 1, mRNA. |
| -1.2 | 0.106768422 | 0.43501376 | MST1 | Homo sapiens macrophage stimulating 1 (hepatocyte growth factor-like) (MST1), mRNA. |
| 1.1 | 0.233296974 | 0.566766396 | MRTO4 | Homo sapiens mRNA turnover 4 homolog (S. cerevisiae) (MRTO4), mRNA. |
| 1.2 | 0.302609585 | 0.62503312 | C1orf151 | Homo sapiens chromosome 1 open reading frame 151 (C1orf151), mRNA. |
| -1.1 | 0.37483697 | 0.67278301 | FAM43B | Homo sapiens family with sequence similarity 43, member B (FAM43B), mRNA. |
| 1.0 | 0.991799561 | 0.995997658 | HS6ST1 | Homo sapiens heparan sulfate 6-O-sulfotransferase 1 (HS6ST1), mRNA. |
| 1.0 | 0.819012657 | 0.927277996 | RPL11 | Homo sapiens ribosomal protein L11 (RPL11), mRNA. |
| 1.5 | 0.050610908 | 0.351463158 | TMEM50A | Homo sapiens transmembrane protein 50A (TMEM50A), mRNA. |
| -1.2 | 0.311502738 | 0.632738603 | SDHD | Homo sapiens succinate dehydrogenase complex, subunit D, integral membrane protein (SDHD), nuclear gene encoding mitochondrial protein, mRNA. |
| -1.2 | 0.059251805 | 0.366886087 | SH3BGRL3 | Homo sapiens SH3 domain binding glutamic acid-rich protein like 3 (SH3BGRL3), mRNA. |
| 3.1 | 0.006 | 0.233 | MT2A | Homo sapiens metallothionein 2A (MT2A), mRNA. |
| -1.2 | 0.201543079 | 0.536360191 | HMGN2 | Homo sapiens high-mobility group nucleosomal binding domain 2 (HMGN2), mRNA. |
| -1.3 | 0.040178354 | 0.335611771 | TRNP1 | Homo sapiens TMF1-regulated nuclear protein 1 (TRNP1), mRNA. |
| 1.2 | 0.70158015 | 0.867353674 | CCDC72 | Homo sapiens coiled-coil domain containing 72 (CCDC72), mRNA. |
| -1.1 | 0.62998267 | 0.833738141 | ATPIF1 | Homo sapiens ATPase inhibitory factor 1 (ATPIF1), nuclear gene encoding mitochondrial protein, transcript variant 3, mRNA. |
| 1.2 | 0.047127998 | 0.351463158 | RCC1 | Homo sapiens regulator of chromosome condensation 1 (RCC1), transcript variant 1, mRNA. |
| -2.4 | 0.063610728 | 0.374566833 | SNORA73A | Homo sapiens small nucleolar RNA, H/ACA box 73A (SNORA73A), non-coding RNA. |
| 1.3 | 0.042259619 | 0.33818367 | RAB42 | Homo sapiens RAB42, member RAS oncogene family (RAB42), mRNA. |
| -1.1 | 0.799183696 | 0.919553799 | RNU11 | Homo sapiens RNA, U11 small nuclear (RNU11), non-coding RNA. |
| 3.1 | 0.001 | 0.207 | MT1H | Homo sapiens metallothionein 1H (MT1H), mRNA. |
| 1.1 | 0.398077208 | 0.693135534 | HMGB4 | Homo sapiens high-mobility group box 4 (HMGB4), transcript variant 1, mRNA. |
| 1.0 | 0.835414024 | 0.933836699 | RPS27 | Homo sapiens ribosomal protein S27 (RPS27), mRNA. |
| 1.1 | 0.623722919 | 0.830064595 | NDUFS5 | Homo sapiens NADH dehydrogenase (ubiquinone) Fe-S protein 5, 15kDa (NADH-coenzyme Q reductase) (NDUFS5), mRNA. |
| 1.0 | 0.818638824 | 0.927277996 | CAP1 | Homo sapiens CAP, adenylate cyclase-associated protein 1 (yeast) (CAP1), transcript variant 1, mRNA. |
| 1.1 | 0.513896615 | 0.760999239 | SMAP2 | Homo sapiens small ArfGAP2 (SMAP2), mRNA. |
| -1.1 | 0.652317945 | 0.846170924 | YBX1 | Homo sapiens Y box binding protein 1 (YBX1), mRNA. |
| -1.2 | 0.061330851 | 0.370072946 | B4GALT2 | Homo sapiens UDP-Gal:betaGlcNAc beta 1,4- galactosyltransferase, polypeptide 2 (B4GALT2), transcript variant 2, mRNA. |
| 1.2 | 0.289881812 | 0.613241297 | RPS8 | Homo sapiens ribosomal protein S8 (RPS8), mRNA. |
| -1.1 | 0.720072481 | 0.879687622 | SNORD46 | Homo sapiens small nucleolar RNA, C/D box 46 (SNORD46), non-coding RNA. |
| 1.2 | 0.037455949 | 0.334360285 | SNORD38A | Homo sapiens small nucleolar RNA, C/D box 38A (SNORD38A), non-coding RNA. |
| -1.2 | 0.162300998 | 0.4921836 | UQCRH | Cytochrome b-c1 complex subunit 6, mitochondrial gene:ENSG00000173660 |
| 1.2 | 0.408662411 | 0.698140798 | CMPK1 | Homo sapiens cytidine monophosphate (UMP-CMP) kinase 1, cytosolic (CMPK1), transcript variant 1, mRNA. |
| -1.1 | 0.149245594 | 0.473907102 | FOXD2 | Homo sapiens forkhead box D2 (FOXD2), mRNA. |
| 1.1 | 0.519864293 | 0.764477933 | STRADB | Homo sapiens STE20-related kinase adaptor beta (STRADB), mRNA. |
| 1.3 | 0.12633428 | 0.451459666 | BTF3L4 | Homo sapiens basic transcription factor 3-like 4 (BTF3L4), transcript variant 1, mRNA. |
| -1.1 | 0.116811811 | 0.445397675 | DMRTB1 | Homo sapiens DMRT-like family B with proline-rich C-terminal, 1 (DMRTB1), mRNA. |
| -1.1 | 0.155028015 | 0.480238297 | PCSK9 | Homo sapiens proprotein convertase subtilisin/kexin type 9 (PCSK9), mRNA. |
| -1.2 | 0.049880471 | 0.351463158 | FOXD3 | Homo sapiens forkhead box D3 (FOXD3), mRNA. |
| -1.2 | 0.237202657 | 0.569540955 | AK3L1 | Homo sapiens adenylate kinase 3-like 1 (AK3L1), nuclear gene encoding mitochondrial protein, transcript variant 5, mRNA. |
| 1.0 | 0.603103498 | 0.815721291 | IL23R | Homo sapiens interleukin 23 receptor (IL23R), mRNA. |
| -1.1 | 0.464672292 | 0.738258328 | SFRS11 | Homo sapiens splicing factor, arginine/serine-rich 11 (SFRS11), mRNA. |
| 1.3 | 0.078366438 | 0.398624785 | TYW3 | Homo sapiens tRNA-yW synthesizing protein 3 homolog (S. cerevisiae) (TYW3), transcript variant 1, mRNA. |
| 1.1 | 0.37067081 | 0.671231883 | ACADM | Homo sapiens acyl-Coenzyme A dehydrogenase, C-4 to C-12 straight chain (ACADM), nuclear gene encoding mitochondrial protein, transcript variant 1, mRNA. |
| -1.3 | 0.414229304 | 0.702231884 | RABGGTB | Homo sapiens Rab geranylgeranyltransferase, beta subunit (RABGGTB), mRNA. |
| 2.3 | 0.004 | 0.222 | CXCL9 | Homo sapiens chemokine (C-X-C motif) ligand 9 (CXCL9), mRNA. |
| 1.2 | 0.156643098 | 0.483351489 | ST6GALNAC5 | Homo sapiens ST6 (alpha-N-acetyl-neuraminyl-2,3-beta-galactosyl-1, 3)-N-acetylgalactosaminide alpha-2,6-sialyltransferase 5 (ST6GALNAC5), mRNA. |
| -1.1 | 0.646030045 | 0.844583009 | RPL17 | Homo sapiens ribosomal protein L17 (RPL17), transcript variant 2, mRNA. |
| 1.4 | 0.03097011 | 0.322843017 | BXDC5 | Homo sapiens brix domain containing 5 (BXDC5), mRNA. |
| -1.1 | 0.58442852 | 0.803145698 | SH3GLB1 | Homo sapiens SH3-domain GRB2-like endophilin B1 (SH3GLB1), mRNA. |
| 1.1 | 0.273751857 | 0.600036693 | LOC339524 | Homo sapiens hypothetical LOC339524 (LOC339524), transcript variant 5, non-coding RNA. |
| 1.2 | 0.142328307 | 0.465467911 | PKN2 | Homo sapiens protein kinase N2 (PKN2), mRNA. |
| 1.7 | 0.030158734 | 0.320144791 | SNORD21 | Homo sapiens small nucleolar RNA, C/D box 21 (SNORD21), non-coding RNA. |
| 1.1 | 0.637692325 | 0.83887882 | FNBP1L | Homo sapiens formin binding protein 1-like (FNBP1L), transcript variant 1, mRNA. |
| 1.2 | 0.053538028 | 0.352204103 | ABCD3 | Homo sapiens ATP-binding cassette, sub-family D (ALD), member 3 (ABCD3), transcript variant 1, mRNA. |
| 1.1 | 0.222439461 | 0.559897262 | CCDC76 | Homo sapiens coiled-coil domain containing 76 (CCDC76), mRNA. |
| -1.1 | 0.520880617 | 0.765555483 | RTCD1 | Homo sapiens RNA terminal phosphate cyclase domain 1 (RTCD1), transcript variant 1, mRNA. |
| -1.1 | 0.115437548 | 0.444567139 | GPR88 | Homo sapiens G protein-coupled receptor 88 (GPR88), mRNA. |
| -1.0 | 0.817002344 | 0.927277996 | VCAM1 | Homo sapiens vascular cell adhesion molecule 1 (VCAM1), transcript variant 1, mRNA. |
| 1.1 | 0.322918877 | 0.642794721 | RNPC3 | Homo sapiens RNA-binding region (RNP1, RRM) containing 3 (RNPC3), mRNA. |
| -1.3 | 0.197155291 | 0.529928003 | AMY2B | Homo sapiens amylase, alpha 2B (pancreatic) (AMY2B), mRNA. |
| 2.1 | 0.004 | 0.222 | S100A8 | Homo sapiens S100 calcium binding protein A8 (S100A8), mRNA. |
| 1.2 | 0.270780394 | 0.596733706 | NBPF6 | Homo sapiens neuroblastoma breakpoint family, member 6 (NBPF6), transcript variant 1, mRNA. |
| 1.0 | 0.982110831 | 0.992445544 | RPL17 | Homo sapiens ribosomal protein L17 (RPL17), transcript variant 2, mRNA. |
| -1.1 | 0.555057888 | 0.782811947 | GNAI3 | Homo sapiens guanine nucleotide binding protein (G protein), alpha inhibiting activity polypeptide 3 (GNAI3), mRNA. |
| 1.2 | 0.352644098 | 0.662085879 | CD53 | Homo sapiens CD53 molecule (CD53), transcript variant 2, mRNA. |
| 2.0 | 0.001 | 0.207 | MT1G | Homo sapiens metallothionein 1G (MT1G), mRNA. |
| 1.1 | 0.241944085 | 0.572466366 | ATP5F1 | Homo sapiens ATP synthase, H+ transporting, mitochondrial F0 complex, subunit B1 (ATP5F1), nuclear gene encoding mitochondrial protein, mRNA. |
| -1.1 | 0.225668748 | 0.560075379 | CAPZA1 | Homo sapiens capping protein (actin filament) muscle Z-line, alpha 1 (CAPZA1), mRNA. |
| -1.0 | 0.778133676 | 0.909928273 | FAM46C | Homo sapiens family with sequence similarity 46, member C (FAM46C), mRNA. |
| -1.0 | 0.820204199 | 0.927595516 | FAM72D | Homo sapiens GCUD2 mRNA for Gastric cancer up-regulated-2, complete cds. |
| 1.2 | 0.473130216 | 0.742619938 | HIST2H2BA | Homo sapiens histone cluster 2, H2ba (HIST2H2BA), non-coding RNA. |
| -1.0 | 0.822322433 | 0.928365516 | SRGAP2 | Homo sapiens SLIT-ROBO Rho GTPase activating protein 2 (SRGAP2), transcript variant 1, mRNA. |
| -1.3 | 0.164786709 | 0.495925055 | NBPF10 | Homo sapiens neuroblastoma breakpoint family, member 10 (NBPF10), mRNA. |
| 1.0 | 0.952835144 | 0.981435132 | TXNIP | Homo sapiens thioredoxin interacting protein (TXNIP), mRNA. |
| 1.5 | 0.021106576 | 0.307098816 | RBM8A | Homo sapiens RNA binding motif protein 8A, mRNA (cDNA clone IMAGE:4687764), complete cds. |
| -1.1 | 0.677294374 | 0.854992752 | RP11-94I2.2 | Homo sapiens neuroblastoma breakpoint family, member 11-like (LOC200030), mRNA. |
| 1.9 | 0.001 | 0.207 | IGHA1 // IGHA1 | Homo sapiens immunoglobulin heavy constant alpha 1 |
| -1.7 | 0.131236178 | 0.454607291 | GPR89B | Homo sapiens G protein-coupled receptor 89B (GPR89B), mRNA. |
| -1.1 | 0.576493006 | 0.79673194 | C1orf152 | Homo sapiens chromosome 1 open reading frame 152 (C1orf152), non-coding RNA. |
| 1.4 | 0.074273169 | 0.389645161 | NBPF16 | Homo sapiens neuroblastoma breakpoint family, member 16 (NBPF16), mRNA. |
| -1.3 | 0.092330131 | 0.420216123 | NBPF16 | Homo sapiens neuroblastoma breakpoint family, member 16 (NBPF16), mRNA. |
| 1.0 | 0.825904237 | 0.929918836 | DRD5 | Homo sapiens dopamine receptor D5 (DRD5), mRNA. |
| -1.3 | 0.065663126 | 0.380389321 | FCGR1A | Homo sapiens Fc fragment of IgG, high affinity Ia, receptor (CD64) (FCGR1A), mRNA. |
| 1.8 | 0.019 | 0.292 | CXCL10 | Homo sapiens chemokine (C-X-C motif) ligand 10 (CXCL10), mRNA. |

Suppl. Table S4 All genes differentially upregulated in tumor cells of THRLBCL compared to LP cells of typical NLPHL, FDR < 0.3, p < 0.05, Fold change > 1.7
