# Supplementary material for: Nodular Lymphocyte Predominant Hodgkin Lymphoma and T Cell/Histiocyte Rich Large B Cell Lymphoma - Endpoints of a Spectrum of One Disease?
Source: PLoS One. 2013 Nov 11;8(11):e78812. doi: 10.1371/journal.pone.0078812 (PMC3823948; doi:10.1371/journal.pone.0078812)
Supplement: Table S5 — All genes upregulated in LP cells of typical nodular NLPHL compared to GC B cells. (DOC) [file pone.0078812.s007.doc]

| Fold change | p-value | FDR | Gene Symbol | Description |
| --- | --- | --- | --- | --- |
| -1.2 | 0.128313069 | 0.167320945 | RER1 | Homo sapiens RER1 retention in endoplasmic reticulum 1 homolog (S. cerevisiae) (RER1), mRNA. |
| -1.3 | 0.118792493 | 0.156459407 | SLC25A33 | Homo sapiens solute carrier family 25, member 33 (SLC25A33), mRNA. |
| 1.1 | 0.395610642 | 0.451838362 | PGD | Homo sapiens phosphogluconate dehydrogenase (PGD), mRNA. |
| 10.2 | 0.000 | 0.000 | UBD | Homo sapiens ubiquitin D (UBD), mRNA. |
| 6.7 | 0.003 | 0.007 | LYZ | Homo sapiens lysozyme (renal amyloidosis) (LYZ), mRNA. |
| -1.1 | 0.794970408 | 0.824854885 | PRAMEF10 // PRAMEF10 // PRAMEF10 | Homo sapiens PRAME family member 10 (PRAMEF10), mRNA. |
| 3.6 | 0.000 | 0.000 | SNORD13 | Homo sapiens small nucleolar RNA, C/D box 13 (SNORD13), non-coding RNA. |
| 3.5 | 0.011 | 0.020 | SNORD6 | Homo sapiens small nucleolar RNA, C/D box 6 (SNORD6), non-coding RNA. |
| 3.4 | 0.009 | 0.017 | MT2A | Homo sapiens metallothionein 2A (MT2A), mRNA. |
| 3.3 | 0.027 | 0.043 | BDP1 | Homo sapiens B double prime 1, subunit of RNA polymerase III transcription initiation factor IIIB (BDP1), mRNA. |
| 3.1 | 0.012 | 0.021 | HSPA1A | Homo sapiens heat shock 70kDa protein 1A (HSPA1A), mRNA. |
| 3.0 | 0.000 | 0.000 | CD63 | Homo sapiens CD63 molecule (CD63), transcript variant 1, mRNA. |
| 1.1 | 0.419996256 | 0.476404329 | TMEM50A | Homo sapiens transmembrane protein 50A (TMEM50A), mRNA. |
| 3.0 | 0.004 | 0.008 | SNORA74A | Homo sapiens small nucleolar RNA, H/ACA box 74A (SNORA74A), non-coding RNA. |
| -1.1 | 0.541442305 | 0.594509582 | SH3BGRL3 | Homo sapiens SH3 domain binding glutamic acid-rich protein like 3 (SH3BGRL3), mRNA. |
| 2.9 | 0.000 | 0.002 | PTCH2 | Patched homolog 2 gene:ENSG00000117425 |
| -1.2 | 0.262648549 | 0.314204875 | HMGN2 | Homo sapiens high-mobility group nucleosomal binding domain 2 (HMGN2), mRNA. |
| -1.0 | 0.906329831 | 0.920589421 | CCDC72 | Homo sapiens coiled-coil domain containing 72 (CCDC72), mRNA. |
| 2.6 | 0.001 | 0.003 | SPRR2B | Homo sapiens small proline-rich protein 2B (SPRR2B), mRNA. |
| 2.6 | 0.001 | 0.004 | POM121 | Homo sapiens POM121 membrane glycoprotein (rat) (POM121), mRNA. |
| -1.2 | 0.35974575 | 0.414731102 | RNU11 | Homo sapiens RNA, U11 small nuclear (RNU11), non-coding RNA. |
| 2.3 | 0.000 | 0.001 | DEFB109P1B | Homo sapiens defensin, beta 109, pseudogene 1B (DEF109P1B), non-coding RNA. |
| -1.2 | 0.177559102 | 0.222255215 | EPB41 | Homo sapiens erythrocyte membrane protein band 4.1 (elliptocytosis 1, RH-linked) (EPB41), transcript variant 2, mRNA. |
| 1.1 | 0.271634996 | 0.323027693 | ZCCHC17 | Homo sapiens zinc finger, CCHC domain containing 17 (ZCCHC17), mRNA. |
| 2.2 | 0.001 | 0.002 | RNU5E | Homo sapiens RNA, U5E small nuclear (RNU5E), non-coding RNA. |
| 2.2 | 0.001 | 0.002 | AMY2A | Homo sapiens amylase, alpha 2A (pancreatic) (AMY2A), mRNA. |
| 2.2 | 0.003 | 0.006 | SNORA42 | Homo sapiens small nucleolar RNA, H/ACA box 42 (SNORA42), non-coding RNA. |
| 2.2 | 0.001 | 0.003 | hCG_1651160 | Homo sapiens Ssu72 RNA polymerase II CTD phosphatase-like (LOC136157), mRNA. |
| 1.3 | 0.056454281 | 0.081762112 | HMGB4 | Homo sapiens high-mobility group box 4 (HMGB4), transcript variant 1, mRNA. |
| 2.2 | 0.000 | 0.001 | OR2T8 | Homo sapiens olfactory receptor, family 2, subfamily T, member 8 (OR2T8), mRNA. |
| 2.2 | 0.023 | 0.038 | SNORD116-6 | Homo sapiens small nucleolar RNA, C/D box 116-6 (SNORD116-6), non-coding RNA. |
| -1.2 | 0.197401741 | 0.244839867 | RPS27 | Homo sapiens ribosomal protein S27 (RPS27), mRNA. |
| 2.1 | 0.000 | 0.001 | CFB | Homo sapiens complement factor B (CFB), mRNA. |
| 2.1 | 0.037 | 0.056 | FYB | Homo sapiens FYN binding protein (FYB-120/130) (FYB), transcript variant 1, mRNA. |
| 2.1 | 0.013 | 0.023 | LRRC37A2 | Homo sapiens leucine rich repeat containing 37, member A2 (LRRC37A2), mRNA. |
| 2.1 | 0.003 | 0.007 | TBC1D3P2 | Homo sapiens TBC1 domain family, member 3 pseudogene 2 (TBC1D3P2), non-coding RNA. |
| 2.1 | 0.003 | 0.007 | FYB | Homo sapiens FYN binding protein (FYB-120/130) (FYB), transcript variant 1, mRNA. |
| 2.0 | 0.026 | 0.042 | SPDYE8P | Homo sapiens speedy homolog E8 (Xenopus laevis), pseudogene (SPDYE8P), non-coding RNA. |
| 2.0 | 0.001 | 0.003 | SLAMF7 | Homo sapiens SLAM family member 7 (SLAMF7), mRNA. |
| 2.0 | 0.040 | 0.060 | ENPP2 | Homo sapiens ectonucleotide pyrophosphatase/phosphodiesterase 2 (ENPP2), transcript variant 1, mRNA. |
| 1.6 | 0.123090837 | 0.161553689 | SNORD46 | Homo sapiens small nucleolar RNA, C/D box 46 (SNORD46), non-coding RNA. |
| 2.0 | 0.001 | 0.003 | GZMK | Homo sapiens granzyme K (granzyme 3; tryptase II) (GZMK), mRNA. |
| 2.0 | 0.002 | 0.005 | C1QTNF9 | Homo sapiens C1q and tumor necrosis factor related protein 9 (C1QTNF9), mRNA. |
| -1.2 | 0.252281618 | 0.303231519 | CMPK1 | Homo sapiens cytidine monophosphate (UMP-CMP) kinase 1, cytosolic (CMPK1), transcript variant 1, mRNA. |
| 2.0 | 0.001 | 0.003 | CFB | Homo sapiens complement factor B (CFB), mRNA. |
| -1.4 | 0.065481574 | 0.092669667 | BTF3L4 | Homo sapiens basic transcription factor 3-like 4 (BTF3L4), transcript variant 1, mRNA. |
| 2.0 | 0.024 | 0.039 | SNORA65 | Homo sapiens small nucleolar RNA, H/ACA box 65 (SNORA65), non-coding RNA. |
| 1.9 | 0.003 | 0.007 | HBII-52-24 | Homo sapiens HBII-52-24 snoRNA (HBII-52-24), non-coding RNA. |
| 1.9 | 0.000 | 0.001 | RNF185 | Homo sapiens ring finger protein 185 (RNF185), transcript variant 4, transcribed RNA. |
| 1.9 | 0.048 | 0.071 | PLA2G7 | Homo sapiens phospholipase A2, group VII (platelet-activating factor acetylhydrolase, plasma) (PLA2G7), mRNA. |
| 1.9 | 0.000 | 0.001 | UBD | Homo sapiens ubiquitin D (UBD), mRNA. |
| 1.9 | 0.013 | 0.023 | SNORA16B | Homo sapiens small nucleolar RNA, H/ACA box 16B (SNORA16B), non-coding RNA. |
| 1.9 | 0.006 | 0.012 | SNORD51 | Homo sapiens small nucleolar RNA, C/D box 51 (SNORD51), non-coding RNA. |
| 1.9 | 0.002 | 0.006 | CXCL13 | Homo sapiens chemokine (C-X-C motif) ligand 13 (CXCL13), mRNA. |
| 1.9 | 0.000 | 0.000 | OR1S2 | Homo sapiens olfactory receptor, family 1, subfamily S, member 2 (OR1S2), mRNA. |
| -1.0 | 0.984801723 | 0.986614877 | NEXN | Homo sapiens nexilin (F actin binding protein) (NEXN), mRNA. |
| 1.8 | 0.000 | 0.001 | MBD3L2 | Homo sapiens methyl-CpG binding domain protein 3-like 2 (MBD3L2), mRNA. |
| 1.8 | 0.018 | 0.030 | LOC100293539 | PREDICTED: Homo sapiens similar to ribosomal protein 10 (LOC100293539), mRNA. |
| 1.8 | 0.000 | 0.001 | PRDM7 | Homo sapiens PR domain containing 7 (PRDM7), transcript variant 1, mRNA. |
| 1.2 | 0.114637396 | 0.151879597 | LOC339524 | Homo sapiens hypothetical LOC339524 (LOC339524), transcript variant 5, non-coding RNA. |
| 1.8 | 0.001 | 0.003 | FAM75A7 | Homo sapiens family with sequence similarity 75, member A7 (FAM75A7), mRNA. |
| 1.8 | 0.000 | 0.001 | STAT1 | Homo sapiens signal transducer and activator of transcription 1, 91kDa (STAT1), transcript variant alpha, mRNA. |

Suppl. Table S5 All genes upregulated in LP cells of typical nodular NLPHL compared to GC B cells (p < 0.05, FDR < 0.1, Fold change > 1.7).
