# Supplementary material for: Nodular Lymphocyte Predominant Hodgkin Lymphoma and T Cell/Histiocyte Rich Large B Cell Lymphoma - Endpoints of a Spectrum of One Disease?
Source: PLoS One. 2013 Nov 11;8(11):e78812. doi: 10.1371/journal.pone.0078812 (PMC3823948; doi:10.1371/journal.pone.0078812)
Supplement: Table S6 — All genes upregulated in LP cells of NLPHL pattern E compared to GC B cells. (DOC) [file pone.0078812.s008.doc]

| Fold change | p-value | FDR | Gene Symbol | mRna - Description |
| --- | --- | --- | --- | --- |
| -1.2 | 0.52712268 | 0.654114753 | RER1 | Homo sapiens RER1 retention in endoplasmic reticulum 1 homolog (S. cerevisiae) (RER1), mRNA. |
| 1.1 | 0.223103047 | 0.350542824 | TPRG1L | Homo sapiens tumor protein p63 regulated 1-like (TPRG1L), mRNA. |
| -1.5 | 0.078784858 | 0.164274005 | PARK7 | Homo sapiens Parkinson disease (autosomal recessive, early onset) 7 (PARK7), transcript variant 1, mRNA. |
| -1.3 | 0.294518577 | 0.427628747 | SLC25A33 | Homo sapiens solute carrier family 25, member 33 (SLC25A33), mRNA. |
| 1.4 | 0.097961216 | 0.193821985 | PGD | Homo sapiens phosphogluconate dehydrogenase (PGD), mRNA. |
| 10.6 | 0.001 | 0.010 | LYZ | Homo sapiens lysozyme (renal amyloidosis) (LYZ), mRNA. |
| 1.2 | 0.158001316 | 0.275201488 | ANGPTL7 | Homo sapiens angiopoietin-like 7 (ANGPTL7), mRNA. |
| 8.9 | 0.004 | 0.020 | UBD | Homo sapiens ubiquitin D (UBD), mRNA. |
| -1.1 | 0.717653627 | 0.808012404 | PRAMEF10 // PRAMEF10 // PRAMEF10 | Homo sapiens PRAME family member 10 (PRAMEF10), mRNA. |
| 5.1 | 0.003 | 0.017 | MT2A | Homo sapiens metallothionein 2A (MT2A), mRNA. |
| 4.6 | 0.003 | 0.015 | TXN | Homo sapiens thioredoxin (TXN), mRNA. |
| 4.5 | 0.005 | 0.023 | HSPA1A | Homo sapiens heat shock 70kDa protein 1A (HSPA1A), mRNA. |
| 1.1 | 0.336760492 | 0.473122855 | RPS14P3 | Homo sapiens ribosomal protein S14 pseudogene 3, mRNA (cDNA clone IMAGE:6201343). |
| 4.5 | 0.001 | 0.009 | CXCL9 | Homo sapiens chemokine (C-X-C motif) ligand 9 (CXCL9), mRNA. |
| 1.2 | 0.523597692 | 0.650942612 | HS6ST1 | Homo sapiens heparan sulfate 6-O-sulfotransferase 1 (HS6ST1), mRNA. |
| -1.3 | 0.072434229 | 0.153856008 | CDC42 | Homo sapiens cell division cycle 42 (GTP binding protein, 25kDa) (CDC42), transcript variant 2, mRNA. |
| 1.5 | 0.05258492 | 0.121180649 | C1QC | Homo sapiens complement component 1, q subcomponent, C chain (C1QC), transcript variant 1, mRNA. |
| 3.5 | 0.003 | 0.016 | CD63 | Homo sapiens CD63 molecule (CD63), transcript variant 1, mRNA. |
| 3.1 | 0.001 | 0.010 | SLAMF7 | Homo sapiens SLAM family member 7 (SLAMF7), mRNA. |
| -1.8 | 0.087345954 | 0.177266918 | RPL11 | Homo sapiens ribosomal protein L11 (RPL11), mRNA. |
| -1.1 | 0.551740708 | 0.675472059 | RCAN3 | Homo sapiens RCAN family member 3 (RCAN3), mRNA. |
| 2.9 | 0.018 | 0.056 | PLA2G7 | Homo sapiens phospholipase A2, group VII (platelet-activating factor acetylhydrolase, plasma) (PLA2G7), mRNA. |
| -1.2 | 0.050699141 | 0.11789929 | TMEM50A | Homo sapiens transmembrane protein 50A (TMEM50A), mRNA. |
| -1.1 | 0.667342407 | 0.768320684 | SDHD | Homo sapiens succinate dehydrogenase complex, subunit D, integral membrane protein (SDHD), nuclear gene encoding mitochondrial protein, mRNA. |
| 2.8 | 0.010 | 0.037 | GBP1 | Homo sapiens guanylate binding protein 1, interferon-inducible, 67kDa (GBP1), mRNA. |
| 1.0 | 0.938483006 | 0.962905322 | HMGN2 | Homo sapiens high-mobility group nucleosomal binding domain 2 (HMGN2), mRNA. |
| 1.4 | 0.432502097 | 0.567552676 | CCDC72 | Homo sapiens coiled-coil domain containing 72 (CCDC72), mRNA. |
| 2.8 | 0.006 | 0.028 | WARS | Homo sapiens tryptophanyl-tRNA synthetase (WARS), transcript variant 1, mRNA. |
| -1.0 | 0.923032531 | 0.950876981 | PHACTR4 | Homo sapiens phosphatase and actin regulator 4 (PHACTR4), transcript variant 1, mRNA. |
| -1.3 | 0.367426595 | 0.50469946 | SNORA73A | Homo sapiens small nucleolar RNA, H/ACA box 73A (SNORA73A), non-coding RNA. |
| 1.2 | 0.292225929 | 0.425153926 | RAB42 | Homo sapiens RAB42, member RAS oncogene family (RAB42), mRNA. |
| -1.4 | 0.217877081 | 0.344488844 | RNU11 | Homo sapiens RNA, U11 small nuclear (RNU11), non-coding RNA. |
| 2.7 | 0.026 | 0.074 | NCRNA00152 | Homo sapiens non-protein coding RNA 152 (NCRNA00152), transcript variant 1, non-coding RNA. |
| 2.6 | 0.013 | 0.046 | GBP5 | Homo sapiens guanylate binding protein 5 (GBP5), transcript variant 1, mRNA. |
| 2.6 | 0.000 | 0.003 | SNORD116-6 | Homo sapiens small nucleolar RNA, C/D box 116-6 (SNORD116-6), non-coding RNA. |
| 2.6 | 0.010 | 0.038 | STAT1 | Homo sapiens signal transducer and activator of transcription 1, 91kDa (STAT1), transcript variant alpha, mRNA. |
| 2.5 | 0.001 | 0.007 | UBD | Homo sapiens ubiquitin D (UBD), mRNA. |
| 2.4 | 0.022 | 0.065 | SUB1 | Homo sapiens SUB1 homolog (S. cerevisiae) (SUB1), mRNA. |
| 2.3 | 0.024 | 0.070 | LOC80154 | Homo sapiens hypothetical LOC80154 (LOC80154), non-coding RNA. |
| -1.3 | 0.103665998 | 0.201976385 | THRAP3 | Homo sapiens thyroid hormone receptor associated protein 3 (THRAP3), mRNA. |
| -1.2 | 0.476153032 | 0.60826972 | RPS27 | Homo sapiens ribosomal protein S27 (RPS27), mRNA. |
| -1.2 | 0.126987787 | 0.234859716 | AKIRIN1 | Homo sapiens akirin 1 (AKIRIN1), transcript variant 1, mRNA. |
| -1.7 | 0.075741965 | 0.159230685 | NDUFS5 | Homo sapiens NADH dehydrogenase (ubiquinone) Fe-S protein 5, 15kDa (NADH-coenzyme Q reductase) (NDUFS5), mRNA. |
| -2.1 | 0.062025962 | 0.136690345 | CAP1 | Homo sapiens CAP, adenylate cyclase-associated protein 1 (yeast) (CAP1), transcript variant 1, mRNA. |
| 2.2 | 0.030 | 0.082 | GPNMB | Homo sapiens glycoprotein (transmembrane) nmb (GPNMB), transcript variant 1, mRNA. |
| -1.7 | 0.053745231 | 0.123069202 | SMAP2 | Homo sapiens small ArfGAP2 (SMAP2), mRNA. |
| -1.2 | 0.313318197 | 0.447730155 | PPIH | Homo sapiens peptidylprolyl isomerase H (cyclophilin H) (PPIH), mRNA. |
| -1.1 | 0.803817785 | 0.873504819 | YBX1 | Homo sapiens Y box binding protein 1 (YBX1), mRNA. |
| 1.3 | 0.086382595 | 0.175887942 | FAM183A | Homo sapiens family with sequence similarity 183, member A (FAM183A), mRNA. |
| 2.2 | 0.018 | 0.056 | CTSB | Homo sapiens cathepsin B (CTSB), transcript variant 2, mRNA. |
| 1.1 | 0.663970461 | 0.765454516 | SNORD46 | Homo sapiens small nucleolar RNA, C/D box 46 (SNORD46), non-coding RNA. |
| 1.0 | 0.901356408 | 0.936331196 | TMEM69 | Homo sapiens transmembrane protein 69 (TMEM69), mRNA. |
| -1.4 | 0.061258913 | 0.135412796 | UQCRH | Cytochrome b-c1 complex subunit 6, mitochondrial gene:ENSG00000173660 |
| -1.0 | 0.834565995 | 0.895054703 | CMPK1 | Homo sapiens cytidine monophosphate (UMP-CMP) kinase 1, cytosolic (CMPK1), transcript variant 1, mRNA. |
| -1.1 | 0.660832783 | 0.763016494 | STRADB | Homo sapiens STE20-related kinase adaptor beta (STRADB), mRNA. |
| 2.2 | 0.025 | 0.072 | CFB | Homo sapiens complement factor B (CFB), mRNA. |
| -1.3 | 0.499943055 | 0.630122938 | BTF3L4 | Homo sapiens basic transcription factor 3-like 4 (BTF3L4), transcript variant 1, mRNA. |
| 2.2 | 0.006 | 0.026 | FYB | Homo sapiens FYN binding protein (FYB-120/130) (FYB), transcript variant 1, mRNA. |
| 2.1 | 0.014 | 0.048 | ADAMDEC1 | Homo sapiens ADAM-like, decysin 1 (ADAMDEC1), transcript variant 1, mRNA. |
| 2.1 | 0.038 | 0.097 | PSAP | Homo sapiens prosaposin (PSAP), transcript variant 1, mRNA. |
| 2.1 | 0.011 | 0.040 | PDXDC2 | Homo sapiens pyridoxal-dependent decarboxylase domain containing 2 (PDXDC2), non-coding RNA. |
| 2.1 | 0.014 | 0.047 | GZMA | Homo sapiens granzyme A (granzyme 1, cytotoxic T-lymphocyte-associated serine esterase 3) (GZMA), mRNA. |
| -1.4 | 0.097789923 | 0.193571382 | TYW3 | Homo sapiens tRNA-yW synthesizing protein 3 homolog (S. cerevisiae) (TYW3), transcript variant 1, mRNA. |
| 1.2 | 0.110593645 | 0.211670185 | LHX8 | Homo sapiens LIM homeobox 8 (LHX8), mRNA. |
| 2.0 | 0.013 | 0.047 | S100A11 | Homo sapiens S100 calcium binding protein A11 (S100A11), mRNA. |
| 2.0 | 0.034 | 0.089 | TBC1D3P2 | Homo sapiens TBC1 domain family, member 3 pseudogene 2 (TBC1D3P2), non-coding RNA. |
| -1.5 | 0.168502391 | 0.289194645 | SNORD45B | Homo sapiens small nucleolar RNA, C/D box 45B (SNORD45B), non-coding RNA. |
| -1.5 | 0.193739095 | 0.319000103 | RPL17 | Homo sapiens ribosomal protein L17 (RPL17), transcript variant 2, mRNA. |
| 2.0 | 0.016 | 0.053 | SNORD13 | Homo sapiens small nucleolar RNA, C/D box 13 (SNORD13), non-coding RNA. |
| 1.9 | 0.000 | 0.001 | S100A4 | Homo sapiens S100 calcium binding protein A4 (S100A4), transcript variant 2, mRNA. |
| 1.9 | 0.000 | 0.003 | NUPR1 | Homo sapiens nuclear protein, transcriptional regulator, 1 (NUPR1), transcript variant 1, mRNA. |
| 1.1 | 0.474444727 | 0.606948072 | LOC339524 | Homo sapiens hypothetical LOC339524 (LOC339524), transcript variant 5, non-coding RNA. |
| 1.9 | 0.016 | 0.054 | FTL | Homo sapiens ferritin, light polypeptide (FTL), mRNA. |
| 1.9 | 0.004 | 0.021 | GZMK | Homo sapiens granzyme K (granzyme 3; tryptase II) (GZMK), mRNA. |
| 1.9 | 0.019 | 0.059 | APOC1 | Homo sapiens apolipoprotein C-I (APOC1), mRNA. |
| -1.2 | 0.361911187 | 0.498922065 | RPL5 | Homo sapiens ribosomal protein L5 (RPL5), mRNA. |
| 1.9 | 0.023 | 0.067 | GIMAP4 | Homo sapiens GTPase, IMAP family member 4 (GIMAP4), mRNA. |
| 1.9 | 0.025 | 0.072 | LGALS2 | Homo sapiens lectin, galactoside-binding, soluble, 2 (LGALS2), mRNA. |
| 1.9 | 0.033 | 0.087 | CXCL13 | Homo sapiens chemokine (C-X-C motif) ligand 13 (CXCL13), mRNA. |
| 1.8 | 0.017 | 0.056 | IL6ST | Homo sapiens interleukin 6 signal transducer (gp130, oncostatin M receptor) (IL6ST), transcript variant 1, mRNA. |
| 1.8 | 0.010 | 0.038 | KRTAP4-9 | Homo sapiens keratin associated protein 4-9 (KRTAP4-9), mRNA. |
| 1.8 | 0.000 | 0.004 | RNU5E | Homo sapiens RNA, U5E small nuclear (RNU5E), non-coding RNA. |

Suppl. Table S6 All genes upregulated in LP cells of NLPHL pattern E compared to GC B cells (p < 0.05, FDR < 0.1, Fold change > 1.7).
