# Supplementary material for: Nodular Lymphocyte Predominant Hodgkin Lymphoma and T Cell/Histiocyte Rich Large B Cell Lymphoma - Endpoints of a Spectrum of One Disease?
Source: PLoS One. 2013 Nov 11;8(11):e78812. doi: 10.1371/journal.pone.0078812 (PMC3823948; doi:10.1371/journal.pone.0078812)
Supplement: Table S7 — All genes upregulated in tumor cells of THRLBCL compared to GC B cells. (DOC) [file pone.0078812.s009.doc]

| Fold change | p-value | FDR | Gene Symbol | mRna - Description |
| --- | --- | --- | --- | --- |
| 1.1 | 0.40539592 | 0.515537044 | KLHL17 | Homo sapiens kelch-like 17 (Drosophila) (KLHL17), mRNA. |
| 1.2 | 0.134502242 | 0.21822416 | PLEKHN1 | Homo sapiens pleckstrin homology domain containing, family N member 1 (PLEKHN1), transcript variant 1, mRNA. |
| 1.1 | 0.330942239 | 0.438399921 | TTLL10 | Homo sapiens tubulin tyrosine ligase-like family, member 10 (TTLL10), transcript variant 2, mRNA. |
| 1.0 | 0.88877337 | 0.925760786 | VWA1 | Homo sapiens von Willebrand factor A domain containing 1 (VWA1), transcript variant 1, mRNA. |
| 1.2 | 0.184003313 | 0.278839143 | MIB2 | Homo sapiens mindbomb homolog 2 (Drosophila) (MIB2), mRNA. |
| 1.1 | 0.246496282 | 0.349618791 | MMP23B | Homo sapiens matrix metallopeptidase 23B (MMP23B), mRNA. |
| -1.1 | 0.27440832 | 0.380146535 | RER1 | Homo sapiens RER1 retention in endoplasmic reticulum 1 homolog (S. cerevisiae) (RER1), mRNA. |
| 1.2 | 0.064184322 | 0.121258121 | C1orf93 | Homo sapiens unknown protein mRNA, complete cds. |
| 1.2 | 0.290462883 | 0.397434637 | ESPN | Homo sapiens espin (ESPN), mRNA. |
| -1.5 | 0.060106968 | 0.114913023 | PARK7 | Homo sapiens Parkinson disease (autosomal recessive, early onset) 7 (PARK7), transcript variant 1, mRNA. |
| 12.2 | 0.000 | 0.000 | UBD | Homo sapiens ubiquitin D (UBD), mRNA. |
| -1.1 | 0.708979989 | 0.787310967 | SLC25A33 | Homo sapiens solute carrier family 25, member 33 (SLC25A33), mRNA. |
| 1.2 | 0.203623027 | 0.301500841 | PGD | Homo sapiens phosphogluconate dehydrogenase (PGD), mRNA. |
| 10.5 | 0.000 | 0.000 | MT2A | Homo sapiens metallothionein 2A (MT2A), mRNA. |
| 6.3 | 0.003 | 0.011 | LYZ | Homo sapiens lysozyme (renal amyloidosis) (LYZ), mRNA. |
| -1.2 | 0.37336587 | 0.482602271 | PRAMEF10 // PRAMEF10 // PRAMEF10 | Homo sapiens PRAME family member 10 (PRAMEF10), mRNA. |
| 3.9 | 0.001 | 0.005 | CXCL9 | Homo sapiens chemokine (C-X-C motif) ligand 9 (CXCL9), mRNA. |
| 1.1 | 0.275031885 | 0.380801928 | SLC25A34 | Homo sapiens solute carrier family 25, member 34 (SLC25A34), mRNA. |
| 1.2 | 0.062426144 | 0.118637502 | C1orf144 | Homo sapiens chromosome 1 open reading frame 144 (C1orf144), transcript variant 1, mRNA. |
| 1.0 | 0.940836086 | 0.963820067 | MST1 | Homo sapiens macrophage stimulating 1 (hepatocyte growth factor-like) (MST1), mRNA. |
| 3.6 | 0.046 | 0.093 | TXN | Homo sapiens thioredoxin (TXN), mRNA. |
| 3.6 | 0.005 | 0.016 | MT1H | Homo sapiens metallothionein 1H (MT1H), mRNA. |
| 1.2 | 0.20311038 | 0.301193576 | FAM43B | Homo sapiens family with sequence similarity 43, member B (FAM43B), mRNA. |
| 1.1 | 0.613792209 | 0.70836024 | HS6ST1 | Homo sapiens heparan sulfate 6-O-sulfotransferase 1 (HS6ST1), mRNA. |
| 3.3 | 0.003 | 0.012 | CD63 | Homo sapiens CD63 molecule (CD63), transcript variant 1, mRNA. |
| 2.8 | 0.001 | 0.005 | SLAMF7 | Homo sapiens SLAM family member 7 (SLAMF7), mRNA. |
| 2.6 | 0.018 | 0.043 | GZMA | Homo sapiens granzyme A (granzyme 1, cytotoxic T-lymphocyte-associated serine esterase 3) (GZMA), mRNA. |
| 2.4 | 0.006 | 0.017 | STAT1 | Homo sapiens signal transducer and activator of transcription 1, 91kDa (STAT1), transcript variant alpha, mRNA. |
| 2.3 | 0.004 | 0.013 | GBP5 | Homo sapiens guanylate binding protein 5 (GBP5), transcript variant 1, mRNA. |
| 2.2 | 0.042 | 0.087 | GBP1 | Homo sapiens guanylate binding protein 1, interferon-inducible, 67kDa (GBP1), mRNA. |
| 2.2 | 0.023 | 0.053 | SNORD13 | Homo sapiens small nucleolar RNA, C/D box 13 (SNORD13), non-coding RNA. |
| 2.1 | 0.004 | 0.014 | FTL | Homo sapiens ferritin, light polypeptide (FTL), mRNA. |
| -1.3 | 0.294025455 | 0.401450288 | TMEM50A | Homo sapiens transmembrane protein 50A (TMEM50A), mRNA. |
| -1.3 | 0.168436572 | 0.26053806 | SDHD | Homo sapiens succinate dehydrogenase complex, subunit D, integral membrane protein (SDHD), nuclear gene encoding mitochondrial protein, mRNA. |
| 1.2 | 0.211819928 | 0.310586838 | GRRP1 | Homo sapiens glycine/arginine rich protein 1 (GRRP1), mRNA. |
| 1.1 | 0.27946262 | 0.385476076 | SH3BGRL3 | Homo sapiens SH3 domain binding glutamic acid-rich protein like 3 (SH3BGRL3), mRNA. |
| 2.1 | 0.013 | 0.034 | MT1G | Homo sapiens metallothionein 1G (MT1G), mRNA. |
| -1.0 | 0.942380641 | 0.964822765 | HMGN2 | Homo sapiens high-mobility group nucleosomal binding domain 2 (HMGN2), mRNA. |
| 2.1 | 0.021 | 0.050 | SNORD34 | Homo sapiens small nucleolar RNA, C/D box 34 (SNORD34), non-coding RNA. |
| 1.1 | 0.465018948 | 0.57188388 | TRNP1 | Homo sapiens TMF1-regulated nuclear protein 1 (TRNP1), mRNA. |
| 1.2 | 0.112954471 | 0.189814279 | GPR3 | Homo sapiens G protein-coupled receptor 3 (GPR3), mRNA. |
| -1.2 | 0.671513741 | 0.757043698 | CCDC72 | Homo sapiens coiled-coil domain containing 72 (CCDC72), mRNA. |
| 2.0 | 0.029 | 0.065 | CFB | Homo sapiens complement factor B (CFB), mRNA. |
| -1.1 | 0.707749627 | 0.786969592 | PHACTR4 | Homo sapiens phosphatase and actin regulator 4 (PHACTR4), transcript variant 1, mRNA. |
| 1.6 | 0.490724937 | 0.596471555 | SNORA73A | Homo sapiens small nucleolar RNA, H/ACA box 73A (SNORA73A), non-coding RNA. |
| 1.2 | 0.136966652 | 0.221536957 | RAB42 | Homo sapiens RAB42, member RAS oncogene family (RAB42), mRNA. |
| -1.2 | 0.716620366 | 0.792355694 | RNU11 | Homo sapiens RNA, U11 small nuclear (RNU11), non-coding RNA. |
| -1.1 | 0.374158169 | 0.483382119 | ZCCHC17 | Homo sapiens zinc finger, CCHC domain containing 17 (ZCCHC17), mRNA. |
| 1.1 | 0.395351248 | 0.505022968 | SERINC2 | Homo sapiens serine incorporator 2 (SERINC2), mRNA. |
| 2.0 | 0.003 | 0.010 | CTSB | Homo sapiens cathepsin B (CTSB), transcript variant 2, mRNA. |
| 2.0 | 0.006 | 0.019 | CFB | Homo sapiens complement factor B (CFB), mRNA. |
| 2.0 | 0.003 | 0.010 | UBD | Homo sapiens ubiquitin D (UBD), mRNA. |
| 2.0 | 0.043 | 0.089 | CXCL10 | Homo sapiens chemokine (C-X-C motif) ligand 10 (CXCL10), mRNA. |
| 1.9 | 0.048 | 0.097 | GZMK | Homo sapiens granzyme K (granzyme 3; tryptase II) (GZMK), mRNA. |
| 1.9 | 0.019 | 0.045 | TBC1D3P2 | Homo sapiens TBC1 domain family, member 3 pseudogene 2 (TBC1D3P2), non-coding RNA. |
| 1.9 | 0.004 | 0.014 | S100A6 | Homo sapiens S100 calcium binding protein A6 (S100A6), mRNA. |
| 1.9 | 0.002 | 0.006 | MS4A6A | Homo sapiens membrane-spanning 4-domains, subfamily A, member 6A (MS4A6A), transcript variant 1, mRNA. |
| 1.8 | 0.027 | 0.060 | ADAMDEC1 | Homo sapiens ADAM-like, decysin 1 (ADAMDEC1), transcript variant 1, mRNA. |
| 1.1 | 0.737884879 | 0.808528388 | TFAP2E | Homo sapiens transcription factor AP-2 epsilon (activating enhancer binding protein 2 epsilon) (TFAP2E), mRNA. |
| 1.8 | 0.035 | 0.076 | GPNMB | Homo sapiens glycoprotein (transmembrane) nmb (GPNMB), transcript variant 1, mRNA. |
| 1.8 | 0.035 | 0.075 | GLUL | Homo sapiens glutamate-ammonia ligase (glutamine synthetase) (GLUL), transcript variant 1, mRNA. |

Suppl. Table S7 All genes upregulated in tumor cells of THRLBCL compared to GC B cells (p < 0.05, FDR < 0.1, Fold change > 1.7).
