# Supplementary material for: Nodular Lymphocyte Predominant Hodgkin Lymphoma and T Cell/Histiocyte Rich Large B Cell Lymphoma - Endpoints of a Spectrum of One Disease?
Source: PLoS One. 2013 Nov 11;8(11):e78812. doi: 10.1371/journal.pone.0078812 (PMC3823948; doi:10.1371/journal.pone.0078812)
Supplement: Methods S1 — (DOC) [file pone.0078812.s010.doc]

**Supplementary Methods and Results**

**Samples and purification**

Microdissection was performed on 5 µm thick RNase free stained HE sections of the frozen tissue samples. Microdissected tumor cells were identified by their typical hyperchromatic and lobulated nuclei. Twohundredtwenty cells were catapulted in PALM adhesive caps and lysed with 2 µl NUGEN Direct Lysis Buffer and further processed according to the NUGEN One-direct protocol.

**Statistical analysis of the microarray data**

Data analysis for quality of the technical performance of the microarrays was performed using the AGCC Viewer (Version 3.0.1.1214) and the Expression Console (Version 1.1) software (Affymetrix). Additionally, the “.DAT” files were visually examined for possible artifacts. Ratios between positive and negative internal controls (positive versus negative area under the curve), number of expressed genes and distribution of expression values were largely comparable across the samples, with similar variations among samples of the different groups, thus indicating that different isolation methods (Laser microdissection or MACS) or slight differences in the RNA quality of live sorted cells (GC B cells) and microdissected tissues did not have a measurable impact on the expression profiling analysis. For the quality control of the arrays, a ratio of the intensities was calculated between positive and negative control probes which showed comparable values for the different arrays. The mean ratio observed was 0.62 (range 0.57 - 0.78). Further statistical analysis was done with the statistical computing environment R. Additional software packages (affy, geneplotter, multtest and vsn) were taken from the Bioconductor project, as previously described.^1^

**Microarray preprocessing.** Probe level normalization was conducted using the variance stabilization method of Huber *et al*..^2^ This method renders the variance of probe intensities approximately independent of their expected expression levels. Parameters (off set and a scaling factor) are estimated for each microarray, assuming that the majority of genes are not differentially expressed across the samples. In view of the computational complexity of the algorithm, parameters are estimated on a random subset of probes and are then used to transform the complete arrays. To compute probe set summaries, for each probe set an additive model on the logarithmic scale (base 2) was fitted to the normalized data of all arrays with the robust median polish method, considering differences in probe affinities via the probe effect.^3^

**Unsupervised hierarchical clustering.** Unsupervised hierarchical clustering was performed for the genes with a standard deviation > 1 across all samples using the Manhattan distance and the mean linkage method.

**Differential gene expression.** Many of the genes on the microarray are not expressed in most of the samples or have only a small variability across the samples. For each pairwise comparison, we first used a global filter to reduce the dimension of the microarray data. We applied an intensity filter (the signal intensity of a probe set should be > 100 in at least 25% of the samples if the group sizes are equal) and a variance filter (the interquartile range of log2 intensities should be at least 0.5 if the group sizes are equal). If the group sizes are not equal, the signal intensity of a probe set should be > 100 in at least a fraction a of the samples, where a is the smaller group size minus one, divided by the total sample size of the two groups. The interquartile range of log2 intensities should be at least 0.1 if the group sizes are not equal. After the global filtering, we applied the two-sample *t* test (assuming equal variance in both groups) to identify genes that are differentially expressed between the two groups. To account for the multiple testing, we used the FDR, as described by Benjamini and Hochberg.^4^ In addition, FC values between the two groups were calculated for each gene. Differentially expressed genes were determined with FDR and FC criteria.

**Principal component analysis.** PCA analysis was performed for the genes with a standard deviation ≥ 1 across all samples.
